# Evidence-based interpretation of uterine cultures in mares: Linking bacterial growth patterns to endometrial inflammation

**DOI:** 10.1371/journal.pone.0356822

**Published:** 2026-08-25

**Authors:** Uxía Yáñez, Milena Krupa, James Gibbons, Julie Storme, Niamh Lewis, Osvaldo Bogado Pascottini

**Affiliations:** 1 School of Veterinary Medicine, University College Dublin, Belfield, Dublin, Ireland; 2 Unit of Reproduction and Obstetrics, Department of Animal Pathology, Faculty of Veterinary Medicine, Universidade de Santiago de Compostela, Lugo, Spain; 3 Irish Equine Centre, Johnstown, Naas, Co. Kildare, Ireland; 4 Waterside Equine Repro Services, Co. Meath, Ireland; University of Saskatchewan Western College of Veterinary Medicine, CANADA

## Abstract

We evaluated uterine bacterial growth patterns and their association with endometrial inflammation (EI) in equine samples collected using uterine swabs (US) or low-volume uterine lavage (UL). A database including 1,545 US and 2,066 UL with cytological and bacteriological results was retrospectively analysed. Endometrial inflammation was defined as ≥2 polymorphonuclear cells per high-power field, and bacterial culture was considered positive when aerobic growth occurred within 48 h, with the number of bacterial isolates per sample recorded (0, 1, 2, or ≥3). Data were analysed using generalized mixed-effects models including the number of isolates and bacterial species as fixed effects. The prevalence of EI was 4.8% for US and 36.6% for UL. For both techniques, EI prevalence was lower in samples with negative bacterial cultures (1.0 ± 0.3% for US and 10.7 ± 1.7% for UL) compared with samples yielding ≥1 bacterial isolate. In UL samples, isolation of a single bacterial species was associated with greater probability of EI (45.1 ± 2.6%) compared with samples yielding ≥3 isolates (33.3 ± 3.1%). For UL, the presence of *Streptococcus* sp. (β-haemolytic) and *Staphylococcus aureus* increased the probability of EI compared with their absence, whereas in US samples only *Streptococcus* sp. (β-haemolytic) increased EI probability. In conclusion, bacterial growth increased the likelihood of EI for both sampling techniques, with *Streptococcus* sp. (β-haemolytic) as the primary bacteria associated with evidence of EI. The integration of endometrial cytology with bacterial culture and pathogen identification improves interpretation of bacteriological findings and supports responsible antimicrobial use in mares.

## Introduction

Endometritis is a significant challenge for the equine breeding industry, not only because it encompasses multiple clinical forms (being classified as acute infectious, chronic infectious, post-mating induced, and chronic degenerative) but also because of its negative impact on fertility [1,2]. Regardless of presentation, affected mares frequently exhibit reduced conception rates, early embryonic loss, and luteal phase abnormalities [3]. Moreover, since antibiotics remain the primary therapeutic approach, accurate and reliable diagnosis is crucial for guiding sustainable antimicrobial stewardship.

Timely and efficient physical clearance of uterine contents after mating or foaling has been recognised as a critical defence mechanism against endometritis, as the ubiquitous presence of potentially pathogenic bacteria represents an underlying risk [1]. Equine endometritis has been previously associated with isolation of potentially pathogenic bacteria including *Escherichia coli*, *Streptococcus* sp., *Staphylococcus* sp., *Klebsiella pneumoniae*, and *Pseudomonas aeruginosa* [4–6], occurring alone, in pairs, or in mixed cultures. However, mixed cultures do not always signify infection, and rather could be a result of contamination either during sampling, transport or plate inoculation. Consequently, bacterial endometritis diagnostic accuracy is markedly improved when cytological assessment of inflammation (polymorphonuclear cells (PMNs) above a certain threshold) is combined with microbial culture of uterine samples, since relying on either technique alone may result in diagnostic misclassification [7,8].

Endometrial samples for the diagnosis of endometritis may be collected using swabs (US) or low-volume lavage (UL). Swabs can be collected either using a double-guarded method or not. Both are simple to perform, although the double guarded method is less prone to contamination [9]. However, all swabbing techniques only collect material from a small focal area of the uterus, which may not reflect the overall uterine health status [10]. In contrast, UL retrieves cells from a larger surface, providing a more representative sample, but the procedure can be more costly and time consuming, and carries greater risk of contamination [3]. In practice, US are often used for both screening and diagnostic purposes, whereas UL are generally reserved for cases of suspected endometritis. When chronic, degenerative endometritis is a concern, endometrial biopsy can provide valuable diagnostic detail. Nevertheless, although considered the “gold standard” for the diagnosis of endometritis, it is an invasive and onerous procedure and requires laborious processing. Therefore, its use is generally limited to particular cases where additional diagnostic certainty is required [8].

The most typical therapeutic approaches for acute and persistent post breeding endometritis include therapeutic uterine flushing, ecbolic agents, anti-inflammatory drugs, and antimicrobial agents, administered either individually or in combination [5]. The use of antimicrobials, however, must be carefully justified, as the global rise in antimicrobial resistance (AMR) poses a serious One Health concern and has prompted stricter regulations, with mandatory antibiogram testing now required in some countries to confirm antibiotic efficacy against the identified pathogens [11]. Consequently, confirming that the species isolated are indeed truly pathogenic and not a result of external contamination (or a commensal member of the uterine microbiome) is crucial to minimize inappropriate use of antibiotics and further contribute to AMR control. In this context, there is ongoing debate regarding the clinical relevance of mixed bacterial growth. Mixed bacterial cultures are frequently considered to represent contamination rather than true uterine infection, particularly when recognised pathogens are not isolated. Supporting this, previous research reported that positive cytological findings were more common in pure compared to mixed cultures, especially in cases involving bacteria such as *Streptococcus* sp. (β-haemolytic) or *E. coli* [12]. These observations raise important questions about whether antimicrobial treatment is justified when mixed bacterial growth is present and highlight the need for studies that evaluate how bacterial load and species composition relate to endometrial inflammation (EI). A clearer understanding of this relationship is essential to support evidence-based therapeutic decisions and reduce unnecessary antibiotic use.

We hypothesized that the number and combination of bacteria isolated from uterine samples have predictive value for EI, as measured by endometrial cytology, and that the isolation of more than three different bacterial species from a single sample can be indicative of contamination. Furthermore, we hypothesized that the technique used to collect uterine samples (US vs. UL) will influence the predictive value of bacterial isolates for endometrial cytology. Therefore, the objective of this large-scale retrospective study was to characterize the associations between single versus mixed bacterial growth and the prevalence of endometrial inflammation in samples collected using either US or UL. Additionally, we aimed to determine which bacterial species, alone or in combination, are most strongly linked to EI.

## Materials and methods

### Data collection

This retrospective observational study was conducted on a dataset including 6,766 endometrial samples (US: n = 4,511; UL: n = 2,255) collected between 2019 and 2024 from mares in Ireland. Samples were obtained by experience veterinary clinicians during routine practice and submitted to an equine veterinary laboratory (Naas, Co. Kildare, Ireland) for diagnostic analysis. All samples were treated according to the laboratory protocols for sample processing. Based on the research objective, we included samples from which both cytology and bacteriological culture data were available, and from which enough epithelial cells to perform an adequate evaluation were collected (US: n = 1,545; UL: n = 2,066). For each sample, information about the year of collection, the farm, the veterinarian, and type of processing was recorded. No individual mare-level information was available, as the laboratory operates under strict confidentiality agreements and cannot disclose client-specific data.

This study was exempted from ethics review, in accordance with the exceptions referred in Article 1 (5.b) of the Directive 2010/63/EU (transposed into national law S.I. No. 543/2012).

### Sample processing and analysis

Samples were received at the laboratory and processed within 4 hours after collection. Uterine swabs were directly used for cytology smear preparation followed by bacterial culture. Uterine lavages were first centrifuged at 3000 RPM for 10 min. The supernatant was discarded, and the sediment was used to prepare both cytologic slides and culture plates.

**Fig 1:**
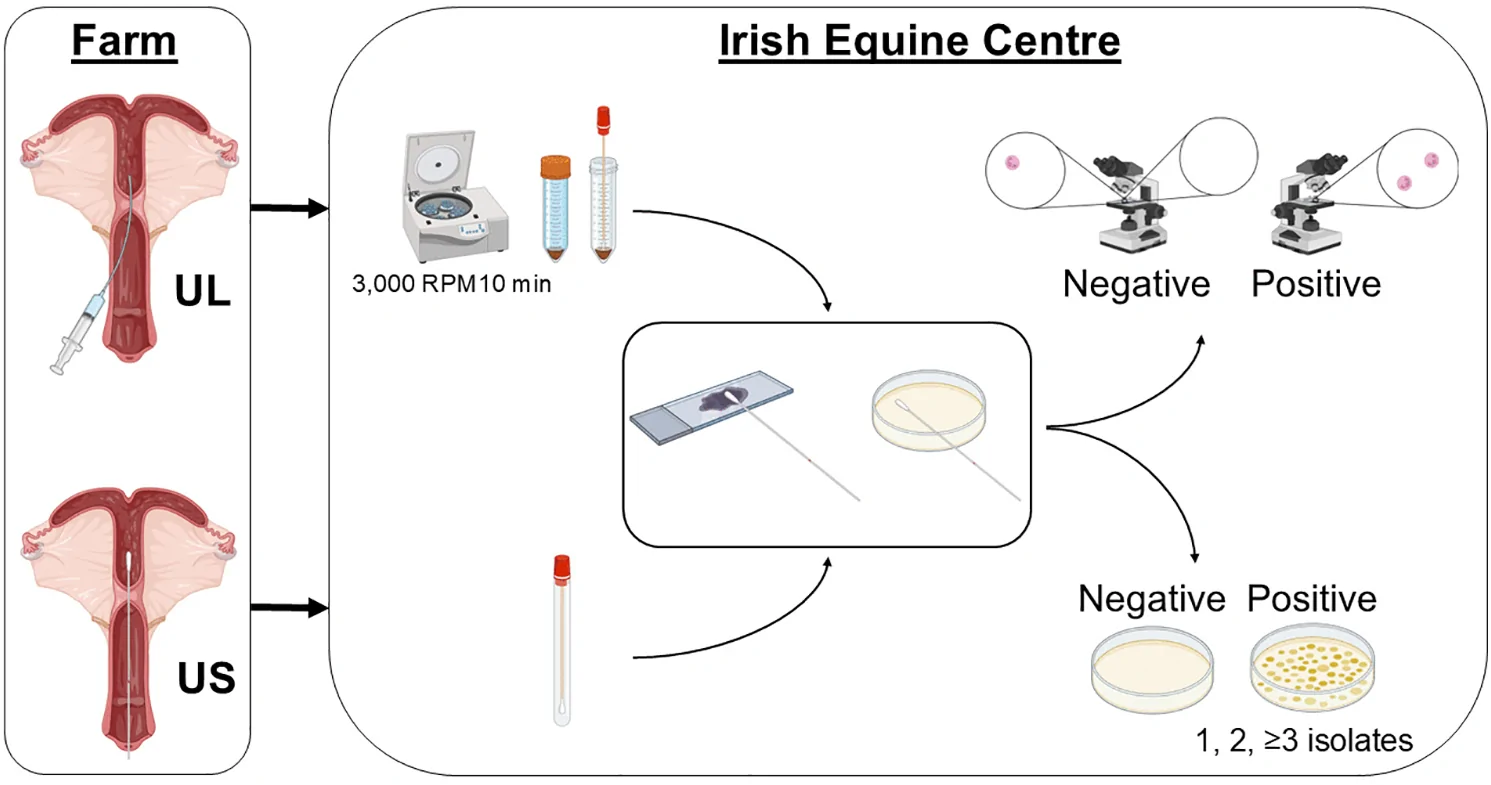
Graphical representation of sample collection in farms and processing at the equine veterinary laboratory of uterine swabs (US) and low-volume uterine lavages (UL) from mares in Ireland. Uterine swabs were rolled onto microscope slides and then directly inoculated into agar plates. Uterine lavages were first centrifuged, the supernatant was removed, and the pellet were scattered onto a microscope slide and inoculated into agar plates using a sterile swab. Samples were considered positive to endometrial inflammation when ≥2 polymorphonuclear neutrophils per high-power field were observed, and negative otherwise. Samples were considered negative to bacterial culture if no growth was observed at 24 and 48 h after culture, and positive to bacterial culture otherwise. Created with Biorender.

For cytological evaluation, US samples and half of the UL pellet were rolled or pipetted onto sterile microscope slides, respectively. The smears were air-dried, stained using Diff-Quik (Fisher Diagnostics, Newark, DE, USA), and examined microscopically at 40× and 100 × magnification by experienced laboratory analysts for the presence of PMNs. Endometrial inflammation was assessed semi-quantitatively based on the occurrence of PMN in at least three randomly selected high-power fields (HPF; 40x magnification). Cytological findings were classified according to the laboratory’s routine scoring system as Negative, Occasional, 1 + , 2 + , 3 + , or 4 + , with increasing scores indicating greater numbers of PMNs and, consequently, a higher degree of inflammation. For this study, samples classified as ‘Negative’ or ‘Occasional’ were considered negative for EI, whereas samples scored as 1 + , 2 + , 3 + , or 4 + were considered positive, corresponding to the presence of ≥2 PMNs/HPF [12]. For bacterial culture, horse blood and MacConkey agar plates (Oxoid, Basingstoke, UK) were inoculated scattered with the US or the sediment of the UL (a sterile swab from the remaining UL pellet was used to inoculate scattered onto the culture plates). Inoculated agar plates were incubated aerobically overnight at 37ºC, checked for bacterial growth at 24 h, then re-incubated and checked again at 48 h. Bacterial identification was carried out via the VITEK 2 system (BioMérieux, Lyon, France) and other biochemical and phenotypic tests where appropriate. Both the number of isolates (0, 1, 2, and ≥3 isolates) and the identity of the bacterial species recovered were recorded. A schematic representation of the sample processing is displayed in Fig 1.

### Statistical analyses

All data was recorded in an Excel file (Microsoft Corporation, Seattle, USA) and organised using the table function. The year, mare, farm of origin (coded), veterinarian (coded), cytology results (positive or negative), bacterial growth and number of isolates (0, 1, 2, ≥ 3), and type of bacteria isolated were considered categorical variables. All statistical analysis were performed in RStudio v.4.4.3. (Posit, Boston, USA). Statistical significance was set at P ≤ 0.05. All analyses were clustered according to the endometrial sampling technique (US or UL) to account for potential differences in diagnostic performance between the two methods.

For each sampling technique (US or UL), bacterial co-occurrence patterns were assessed by identifying pairwise combinations of isolates within samples. The frequency of each pair was retrieved separately for EI-positive and EI-negative samples, and associations with EI status were evaluated using Fisher’s exact test. Additionally, to visualize the structure of bacterial communities, co-occurrence networks were constructed. Only bacterial taxa with a prevalence >1% were included. Networks were generated using the *igraph* package.

To assess the influence of the number of bacterial isolates (0, 1, 2, ≥ 3 isolates) on the probability of EI (yes vs no), generalized mixed-effects models (GLMMs) were fitted using the *glmer* function from the *lme4* package in R for each sampling technique. The number of isolates was included as a fixed effect. To further investigate the influence of specific bacterial taxa, separate GLMMs were fitted for the most prevalent species (≥1%). Each model included the binary presence/absence of the target species as a fixed effect, together with the number of isolates and its interaction. For all the models, mare identity and veterinarian were initially evaluated as random effects; however, their inclusion led to model instability and convergence issues in US data for both effects, and in UL data for the veterinarian effect. Therefore, random effects were specified for mare identity to account for non-independence of repeated observations, and for year nested within farm of origin to account for clustering related to management and temporal effects in UL models, whereas for US models only year nested within farm of origin was retained. The selection of the random-effects structure was based on biological relevance and Akaike Information Criterion. Also, in all models, the estimate marginal means were obtained using the *emmeans* package, and post-hoc pairwise comparisons were performed with Tukey adjustment for multiple testing. Differences between groups were denoted by compact letter display.

Model diagnostics were conducted with the *DHARMa* package to evaluate model assumptions and fit. The models showed adequate fit, with no evidence of overdispersion, zero-inflation, or deviation from the expected distribution of residuals.

## Results

### Descriptive data

**Fig 2:**
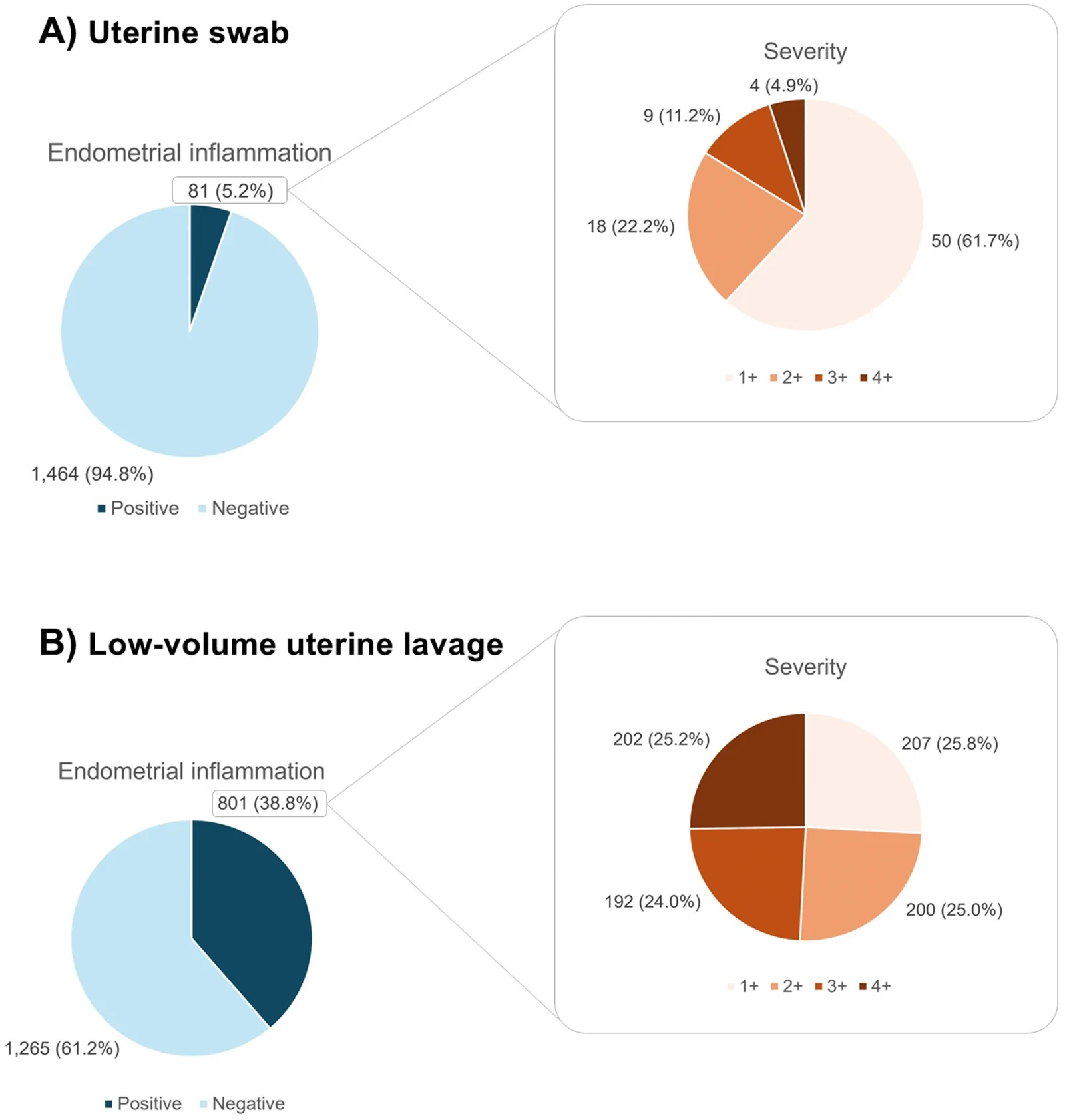
Prevalence and severity of endometrial inflammation (EI) observed for A) uterine swabs (n = 1,545) and B) low-volume uterine lavages (n = 2,066) collected from mares in Ireland. Samples were considered positive to EI if ≥2 polymorphonuclear neutrophils per high-power field (PMN/HPF) were observed, and negative otherwise. Severity was scored semi-quantitatively based on PMN proportion, being 1 + the mildest degree (equivalent to 2 PMN/HPF).

**Fig 3:**
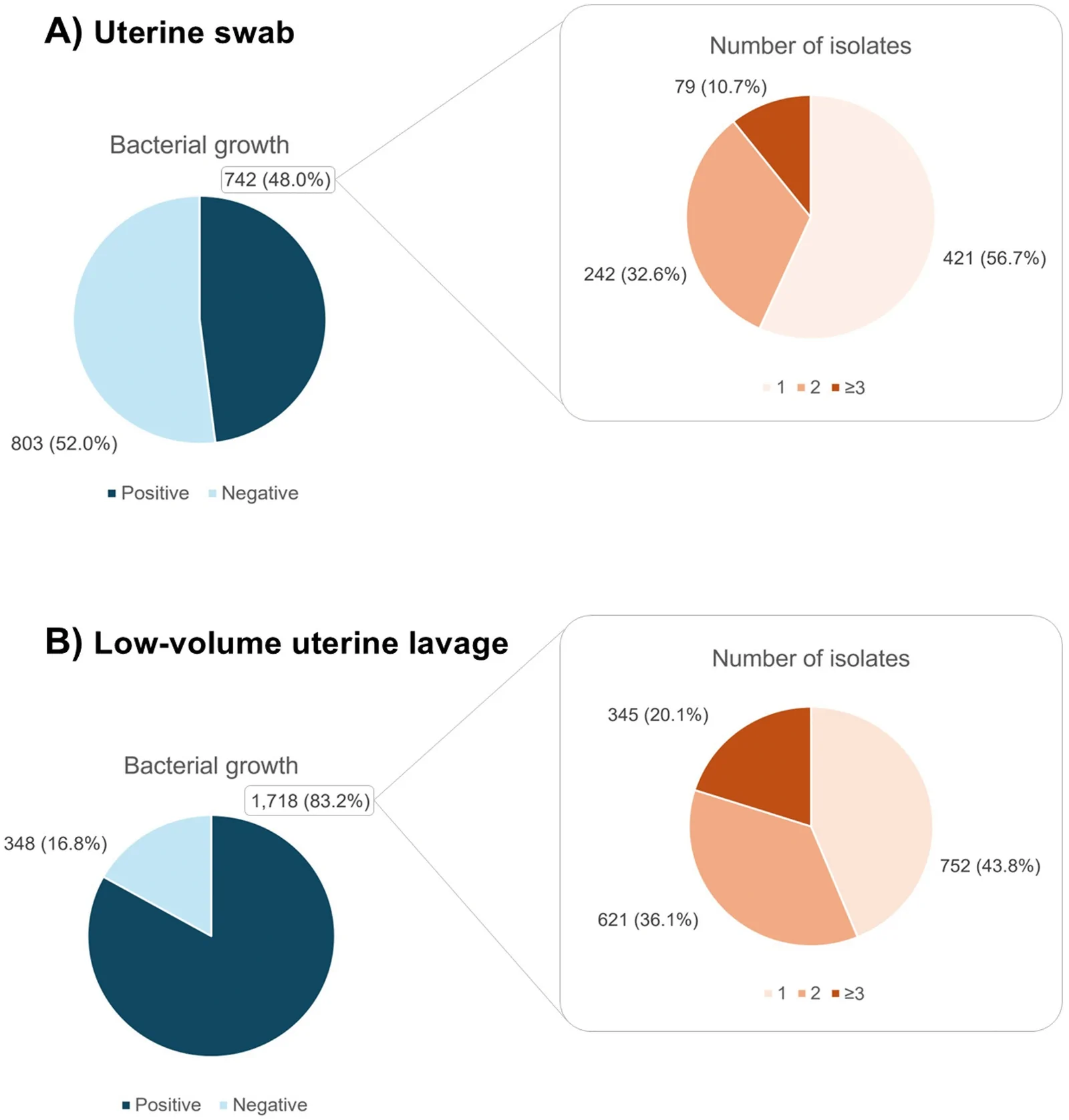
Prevalence of bacterial growth and number of isolates (1, 2, ≥ 3) observed for A) uterine swabs (US; n = 1,545) and B) low-volume uterine lavages (UL; n = 2,066) collected from mares in Ireland. Samples were considered negative to bacterial culture if no growth was observed at 24 and 48 h after culture, and positive otherwise. The bacterial species present were identified, and the number of isolates was recorded.

Results showing the prevalence and severity of EI, and bacterial growth and number of isolates, for US and UL samples are summarized in Figs 2 and 3, respectively. Additionally, the proportion of samples positive or negative to EI and positive or negative to bacterial growth and number of isolates are shown in Table 1 for both sampling techniques. Further detail into the frequency of the different grades for endometrial inflammation and the association with the number of bacterial isolated is described in S1 and S2 Tables.

**Table 1:** Distribution of samples according to cytology and bacterial culture results, accounting for the number of bacterial isolates identified, for uterine swabs (US; n = 1,545) and low-volume uterine lavages (UL; n = 2,066) collected from mares in Ireland.

Endometrial inflammation		Bacterial growth			
		Positive			Negative
		1	2	≥3	0
**US**	**Positive**	37 (45.7%)	22 (27.2%)	13 (16.0%)	9 (11.1%)
	**Negative**	384 (26.2%)	220 (15.0%)	66 (4.5%)	794 (54.3%)
**UL**	**Positive**	366 (45.7%)	263 (32.8%)	125 (15.6%)	47 (5.9%)
	**Negative**	386 (30.5%)	358 (28.3%)	220 (17.4%)	301 (23.8%)

Percentages are calculated based on the total number of samples within each row. Samples were considered positive to endometrial inflammation if ≥2 polymorphonuclear neutrophils per high-power field were observed, and negative otherwise. Samples were considered negative to bacterial culture if no growth was observed at 24 and 48 h after culture, and positive otherwise. 0, 1, 2, and ≥3 refer to the number of isolates obtained per sample.

For positive isolated US samples, the most abundant bacteria (prevalence >1%) were *Streptococcus* sp. (β-haemolytic) (n = 519, 26.5%), *E. coli* (n = 291, 14.8%), *Streptococcus* sp. (α-haemolytic) (n = 124, 6.3%), *Staphylococcus* sp. (n = 55, 2.8%), *Staphylococcus aureus* (n = 46, 2.3%), and *Klebsiella aerogenes* (n = 22, 1.1%).

For positively isolated UL samples, the most abundant bacteria (prevalence >1%) were *Streptococcus* sp. (β-haemolytic) (n = 1221, 35.1%), *E. coli* (n = 907, 26.1%), *Streptococcus* sp. (α-haemolytic) (n = 301, 8.7%), *Staphylococcus* sp. (n = 177, 5.1%), *S. aureus* (n = 128, 3.7%), *K. aerogenes* (n = 78, 2.2%), *Corynebacterium* sp. (n = 48, 1.4%), and *Klebsiella pneumoniae* ssp. *pneumoniae* (n = 47, 1.3%).

**Fig 4:**
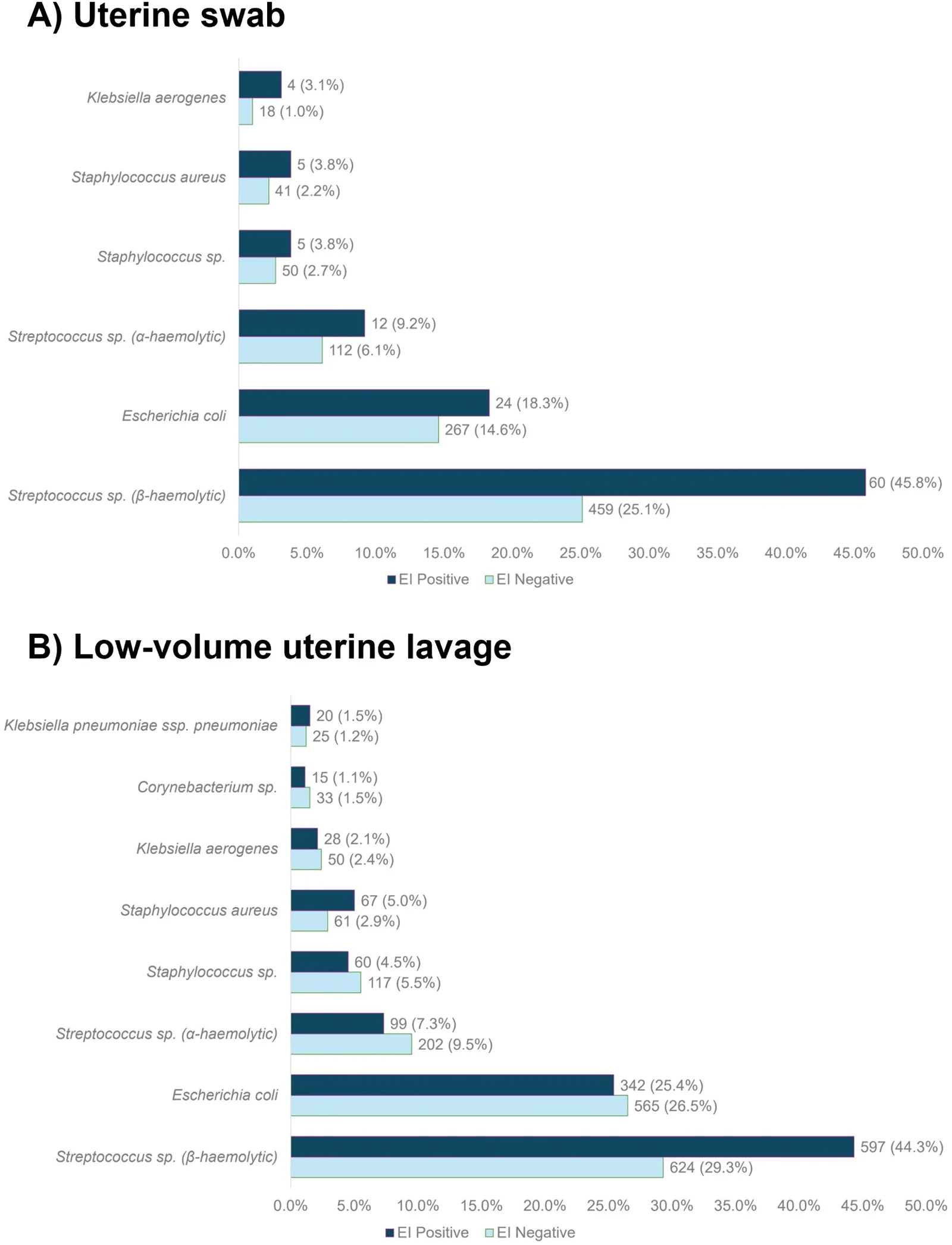
Frequency and proportion of the most common bacterial species (prevalence >1%) isolated from A) uterine swabs (n = 1,545) and B) low-volume uterine lavages (n = 2,066) collected from mares in Ireland, stratified by the diagnosis of endometrial inflammation (EI). Samples were considered positive to EI if ≥2 polymorphonuclear neutrophils per high-power field were observed, and negative otherwise. Proportions are expressed within EI status for each sampling technique.

**Fig 5:**
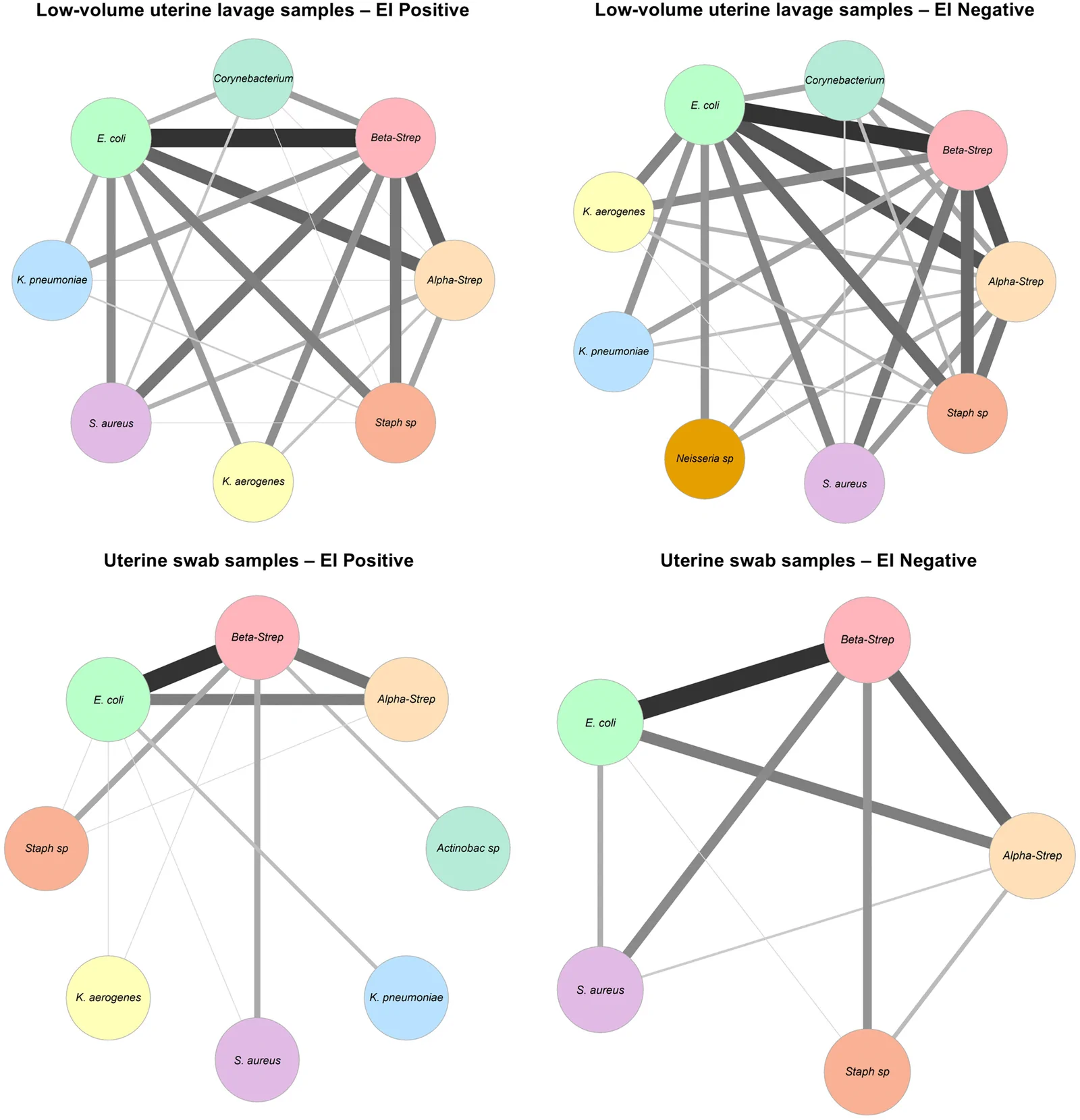
Network plots for the most common bacterial combinations observed in low-volume uterine lavages (UL) and uterine swabs (US) from mares depending on the diagnosis of endometrial inflammation (EI): positive (UL: n = 388; US: n = 35) or negative (UL: n = 578; US: n = 286). The line width and colour intensity reflect the frequency of each bacterial pair, with thicker and darker lines indicating a greater number of co-occurrences. Samples were considered positive to EI if ≥2 polymorphonuclear neutrophils per high-power field were observed, and negative otherwise. Bacterial combinations include the observation of 2 or ≥3 isolates at 24 and 48 h after culture. Beta-Strep: Streptococcus sp. (β-haemolytic); Alpha-Strep: Streptococcus sp. (α-haemolytic). Staph sp: Staphylococcus sp.; Neiss sp: Neisseria sp.; Klebsiella aerogenes; Staphylococcus aureus; Klebsiella pneumoniae ssp. pneumoniae; Escherichia coli; Corynebacterium sp.

The distribution of each bacterium according to the diagnosis of EI for US and UL is represented in Fig 4. Additionally, the most common bacterial combinations found in US and UL, positive or negative to EI, are depicted in Fig 5.

### Bacterial culture characteristics and prediction of endometrial inflammation in uterine swabs

The predicted probability (± standard error [SE]) of EI was greater in samples with positive bacterial culture (1 isolate: 8.8 ± 1.4%; 2 isolates: 9.1 ± 1.9%; ≥ 3 isolates: 16.5 ± 4.2%) compared to negative bacterial culture (1.1 ± 0.4%, *P* < 0.001). However, no differences were detected among the number of isolates (*P* > 0.17). The distribution of the most common bacterial combinations among US samples is shown in S3 Table. Due to the low number occurrences for most pairs, statistical comparisons could not be performed.

Regarding isolated species in the sample, the predicted probability of EI (± SE) was greater in samples containing *Streptococcus* sp. (β-haemolytic) (11.6 ± 1.4% vs. 2.0 ± 0.4%, *P* < 0.001), *E. coli* (8.3 ± 1.6% vs. 4.5 ± 0.5%, *P*  = 0.01), *K. aerogenes* (18.1 ± 8.3% vs. 5.1 ± 0.6%, *P* = 0.01), or *Streptococcus* sp. (α-haemolytic) (9.7 ± 2.7% vs. 4.9 ± 0.6%, *P* = 0.02), than in those where the respective microorganism was absent, independent of the presence of other bacteria. No significant associations were found between EI and the presence/absence of the remaining bacterial species (S4 Table; *P* > 0.09). The predicted probabilities of EI stratified by individual bacterial species (prevalence >1%) and number of isolates are shown in Table 2. In samples where *Streptococcus* sp. (β-haemolytic) was isolated as a single species, the probability of EI was greater compared to samples with the growth of a different bacterium as a single isolate (11.9 ± 2.0% vs. 3.3 ± 1.4%, *P* = 0.004). Unlike the overall prevalence pattern, samples in which *E. coli* occurred as a single isolate exhibited lesser predicted probability of EI than those without this organism, independent of the growth of a different bacterium as single isolate (1.3 ± 1.3% vs. 10.5 ± 1.7%, *P* = 0.02). No differences on the predicted probability of EI were detected for the isolation of the remaining bacterial species (*P* > 0.18).

**Table 2:** Associations between most prevalent (>1%) bacterial species and endometrial inflammation (EI) in uterine swabs from mares, determined by generalized mixed-effects models. For each bacterial species, the table shows the predicted probability (± SE) of EI according to the number of bacterial isolates and the presence (Yes) or absence (No) of the species within each isolate category. Samples were considered positive to EI if ≥2 polymorphonuclear neutrophils per high-power field were observed, and negative otherwise. 1, 2, and ≥3 refer to the number of isolates obtained per sample at 24 and 48 h after culture.

Bacterium	Number of isolates	Species detected	Predicted probability (± SE) of EI (%)	*P*-value
*Streptococcus* sp. (β-haemolytic)	1	No (n = 5)	3.3 ± 1.4	0.004
		Yes (n = 32)	11.9 ± 2.0	
	2	No (n = 6)	9.8 ± 3.8	0.81
		Yes (n = 16)	8.8 ± 2.1	
	≥3	No (n = 1)	11.1 ± 10.5	0.64
		Yes (n = 12)	17.1 ± 4.5	
*Staphylococcus aureus*	1	No (n = 36)	8.7 ± 1.4	0.50
		Yes (n = 1)	16.7 ± 15.2	
	2	No (n = 20)	9.2 ± 2.0	0.89
		Yes (n = 2)	8.3 ± 5.6	
	≥3	No (n = 11)	17.5 ± 4.8	0.63
		Yes (n = 2)	12.5 ± 8.3	
*Escherichia coli*	1	No (n = 36)	10.5 ± 1.7	0.03
		Yes (n = 1)	1.3 ± 1.3	
	2	No (n = 10)	11.1 ± 3.3	0.40
		Yes (n = 12)	7.9 ± 2.2	
	≥3	No (n = 2)	10.0 ± 6.7	0.38
		Yes (n = 11)	18.6 ± 5.1	
*Klebsiella aerogenes*	1	No (n = 36)	8.7 ± 1.4	0.60
		Yes (n = 1)	14.3 ± 13.2	
	2	No (n = 20)	8.6 ± 1.8	0.18
		Yes (n = 2)	22.2 ± 13.9	
	≥3	No (n = 12)	16.4 ± 4.3	0.98
		Yes (n = 1)	16.7 ± 15.2	
*Staphylococcus* sp.^1^	1	No (n = 37)	NA	NA
		Yes (n = 0)	NA	
	2	No (n = 19)	8.7 ± 1.9	0.49
		Yes (n = 3)	13.0 ± 7.0	
	≥3	No (n = 11)	17.2 ± 4.7	0.71
		Yes (n = 2)	13.3 ± 8.8	
*Streptococcus* sp*. (*α-haemolytic)	1	No (n = 36)	9.2 ± 1.5	0.33
		Yes (n = 1)	3.6 ± 3.5	
	2	No (n = 18)	9.7 ± 2.2	0.53
		Yes (n = 4)	7.0 ± 3.4	
	≥3	No (n = 6)	15.0 ± 5.7	0.72
		Yes (n = 7)	18.0 ± 6.2	

^1^Interaction terms between bacterium and the number of isolates were initially explored but led to rank-deficient models due to sparse combinations of predictor levels. Therefore, only additive effects were retained in the final model. SE: standard error. NA: non-applicable.

### Bacterial culture characteristics and prediction of endometrial inflammation in low-volume uterine lavages

The predicted probability (± SE) of EI was greater in samples with positive bacterial culture (1 isolate: 47.5 ± 2.5%; 2 isolates: 41.1 ± 2.6%; ≥ 3 isolates: 35.2 ± 3.1%) compared to negative bacterial culture (12.7 ± 2.0%, *P* < 0.001). When comparing the number of isolates, samples with a single isolate had greater probability of EI than those with ≥3 isolates (*P* = 0.001), while no differences were detected between 1 and 2 isolates (*P* = 0.10) or between 2 and ≥3 isolates (*P* = 0.29).

The distribution of the most common bacterial combinations among UL samples is shown in S5 Table. The pairs *Streptococcus* sp. (β-haemolytic) & *E. coli* and *Streptococcus* sp. (α-haemolytic) & *Staphylococcus* sp. were more frequent in EI-negative compared to EI-positive samples (57.1% vs. 42.9%, *P* = 0.02; 81.0% vs. 19.0%, *P* = 0.001, respectively). No differences were observed for any other combination of microorganisms (*P* > 0.13).

Regarding species isolation, the predicted probability of EI (±SE) was greater in samples containing *Streptococcus* sp. (β-haemolytic) than in those in which it was absent, independent of the presence of other bacteria (48.1 ± 2.1% vs. 23.4 ± 1.9%; *P* < 0.001). Likewise, the presence of *S. aureus* was associated with greater predicted probability of EI compared with samples lacking this pathogen, even when other bacteria were present (51.2 ± 4.9% vs. 36.5 ± 1.9%; *P* = 0.001). In contrast, *Streptococcus* sp. (α-haemolytic) was associated with lesser predicted probability of EI relative to samples without this organism, without regard to the presence of other bacteria (30.6 ± 3.2% vs. 38.6 ± 2.1%; *P* = 0.01). No significant associations were observed between EI and the presence/absence of the remaining bacterial species (S6 Table; *P* > 0.17). The predicted probabilities of EI stratified by individual bacterial species (prevalence >1%) and number of isolates are shown in Table 3. The presence of *Streptococcus* sp. (β-haemolytic) was consistently associated with greater predicted probability of EI when the organism was isolated as a single species (56.3 ± 2.8% vs. 34.7 ± 3.2; *P* < 0.001), 2 isolates (46.8 ± 2.8 vs. 27.5 ± 3.8; *P* < 0.001), or ≥3 isolates (37.7 ± 3.3 vs. 20.9 ± 5.7; *P* = 0.02) compared to samples where this pathogen was not isolated, independent of the growth of other bacteria. Similarly, in samples containing a single isolate, the predicted probability of EI was greater when *S. aureus* was identified than when this pathogen was absent, irrespective of other bacterial presence (79.5 ± 9.3% vs. 46.8 ± 2.4%, *P* = 0.01). On the contrary, the presence of *E. coli* as a single isolate, and *Streptococcus* sp. (α-haemolytic) in samples containing two isolates, was associated with lesser predicted probability of EI compared with samples negative for these organisms, independent of the presence of other bacterial taxa (29.4 ± 3.7 vs. 53.7 ± 2.5, *P* < 0.001; 32.0 ± 4.9 vs. 43.0 ± 2.7, *P* = 0.04, respectively).

**Table 3:** Associations between most prevalent (>1%) bacterial species and endometrial inflammation (EI) in low-volume uterine lavages from mares, determined by generalized mixed-effects models. For each bacterial species, the table shows the predicted probability (± SE) of EI according to the number of bacterial isolates and the presence (Yes) or absence (No) of the species within each isolate category. Samples were considered positive to EI if ≥2 polymorphonuclear neutrophils per high-power field were observed, and negative otherwise. 1, 2, and ≥3 refer to the number of isolates obtained per sample at 24 and 48 h after culture.

Bacterium	Number of isolates	Species detected	Predicted probability (± SE) of EI (%)	*P*-value
*Streptococcus* sp. (β-haemolytic)	1	No (n = 99)	34.7 ± 3.2	< 0.001
		Yes (n = 267)	56.3 ± 2.8	
	2	No (n = 46)	27.5 ± 3.8	< 0.001
		Yes (n = 217)	46.8 ± 2.8	
	≥3	No (n = 12)	20.9 ± 5.7	0.02
		Yes (n = 113)	37.7 ± 3.3	
*Staphylococcus aureus*	1	No (n = 351)	46.8 ± 2.4	0.01
		Yes (n = 15)	79.5 ± 9.3	
	2	No (n = 232)	40.2 ± 2.6	0.09
		Yes (n = 31)	52.0 ± 6.9	
	≥3	No (n = 104)	34.3 ± 3.2	0.45
		Yes (n = 21)	39.9 ± 7.1	
*Escherichia coli*	1	No (n = 314)	53.7 ± 2.5	< 0.001
		Yes (n = 52)	29.4 ± 3.7	
	2	No (n = 91)	40.0 ± 2.7	0.23
		Yes (n = 172)	45.2 ± 3.9	
	≥3	No (n = 17)	29.9 ± 6.4	0.37
		Yes (n = 108)	36.4 ± 3.2	
*Klebsiella pneumoniae* ssp*. pneumoniae*^1^	1	No (n = 366)	NA	NA
		Yes (n = 0)	NA	
	2	No (n = 254)	41.3 ± 2.9	0.16
		Yes (n = 9)	52.8 ± 8.5	
	≥3	No (n = 114)	34.5 ± 3.4	0.16
		Yes (n = 11)	45.6 ± 8.3	
*Klebsiella aerogenes*	1	No (n = 361)	47.7 ± 2.4	0.89
		Yes (n = 5)	49.9 ± 16.4	
	2	No (n = 252)	41.8 ± 2.6	0.29
		Yes (n = 11)	32.3 ± 8.3	
	≥3	No (n = 113)	35.2 ± 3.1	0.95
		Yes (n = 12)	34.6 ± 8.5	
*Staphylococcus* sp.	1	No (n = 360)	48.0 ± 2.4	0.18
		Yes (n = 6)	31.6 ± 11.2	
	2	No (n = 241)	41.3 ± 2.6	0.98
		Yes (n = 22)	41.1 ± 7.1	
	≥3	No (n = 94)	38.0 ± 3.5	0.10
		Yes (n = 31)	28.5 ± 4.7	
*Streptococcus* sp*. (*α-haemolytic)	1	No (n = 361)	48.1 ± 2.5	0.09
		Yes (n = 5)	27.0 ± 10.6	
	2	No (n = 228)	43.0 ± 2.7	0.04
		Yes (n = 35)	32.0 ± 4.9	
	≥3	No (n = 66)	39.6 ± 4.2	0.09
		Yes (n = 59)	30.8 ± 3.8	
*Corynebacterium* sp.	1	No (n = 365)	48.0 ± 2.4	0.16
		Yes (n = 1)	16.0 ± 15.0	
	2	No (n = 259)	41.6 ± 2.5	0.31
		Yes (n = 4)	27.8 ± 12.2	
	≥3	No (n = 115)	35.2 ± 3.1	0.91
		Yes (n = 10)	34.1 ± 9.2	

^1^Interaction terms between bacterium and the number of isolates were initially explored but led to rank-deficient models due to sparse combinations of predictor levels. Therefore, only additive effects were retained in the final model. SE: standard error. NA: non-applicable

## Discussion

This study provides a large-scale retrospective evaluation of EI and bacterial culture outcomes from US and UL samples collected from mares under field conditions in Ireland. Because US and UL samples originated from independent clinical populations and were not collected simultaneously from the same mares, differences observed between datasets should be interpreted descriptively rather than as evidence of differences in diagnostic performance between sampling techniques. Accordingly, all associations reported in this study should be interpreted within each sampling method. The prevalence of EI was 5.2% in US samples and 38.8% in UL samples, with bacterial growth detected in 48.0% and 83.2% of samples, respectively. Within each sampling technique, culture-positive samples showed greater probability of EI compared to culture-negative samples. However, differences related to the number of isolates were only evident in the UL dataset, as samples with a single isolate had greater predicted probability of EI compared to samples with ≥3 isolates. These associations were derived from the adjusted GLMMs analysis and should not be interpreted directly from descriptive frequencies, which do not account for the effects of the number of bacterial isolates or the random-effects structure included in the models. Similar bacterial profiles were identified across both datasets, with *Streptococcus* sp. (β-haemolytic), *E. coli*, *Streptococcus* sp. (α-haemolytic), *Staphylococcus* sp., *S. aureus* and *Klebsiella* sp. being most frequently isolated, and the pairs *Streptococcus* sp. (β-haemolytic) & *E. coli*, *Streptococcus* sp. (α-haemolytic) & *Streptococcus* sp. (β-haemolytic), and *Streptococcus* sp. (α-haemolytic) & *E. coli* being the most common co-occurrences. Notably, *Streptococcus* sp. (β-haemolytic) significantly increased the probability of EI in both sampling methods. Altogether, these results suggest that, independent of the sampling technique, bacteriological findings must always be interpreted in conjunction with cytological results, and that treatment decisions should be guided by this integrated assessment.

The markedly greater prevalence of EI and positive bacterial culture observed in the UL dataset compared with the one obtained in the US dataset is consistent with known differences in the nature of the sampling technique per se and the clinical approach for the selection of the sampling method. Low-volume uterine lavage recovers cellular material from a broader surface area of the endometrium in contrast to the localized sampling achieved by a swab, which may influence the likelihood of detecting inflammatory cells when lesions are focal or when inflammatory cells are unevenly distributed in US sampling [10,13,14]. Additionally, the main use of UL when endometritis is clinically suspected instead of US, which are commonly used for screening purposes before breeding, is also an important factor to consider when interpreting our results [15]. This difference in clinical application is likely to have contributed to the greater EI prevalence observed in UL samples, in agreement with previous studies that also reported low EI prevalence during routine screening sampling [16]. A further methodological consideration relates to sample processing and cytological evaluation. In UL samples, centrifugation and concentration of the cellular pellet may increase the apparent number of inflammatory cells, potentially resulting in greater PMN counts per HPF compared to US preparations. As the same diagnostic threshold (≥2 PMNs/HPF) was applied for both sampling techniques, this may have introduced a degree of bias when directly comparing EI prevalence between US and UL. Alternative approaches, such as using PMN proportions rather than absolute counts, may be more accurate and provide a more standardized assessment across sampling methods and should be considered in future studies.

An important limitation of this study is the absence of individual information of the mares from which the samples were collected. Due to ethical and confidentiality considerations, all samples were anonymized. Moreover, the absence of information regarding oestrous stage represents an additional limitation, as physiological variations in PMN number occur throughout the reproductive cycle. However, reproductive stage would be expected to act as a source of random biological variation more likely to attenuate associations than to generate them. Moreover, as our results are based on the relationship between bacteriological findings and cytological evidence of inflammation, mild neutrophil infiltration during oestrus would not be enough to explain the consistent species-specific associations observed across the large number of samples analysed. Additionally, another limitation would be that we did not directly compare the performance of US and UL, as both samples would ideally need to be collected from the same animal. Therefore, although comparing sampling techniques was not the primary aim of this study, no definitive conclusions can be drawn regarding differences in EI prevalence attributable to the sampling method. Yet, this does not affect the validity of the associations identified within US and UL datasets individually.

Uterine swab and UL sampling revealed a similar distribution of the most commonly isolated bacterial species. Moreover, the pairs *Streptococcus* sp. (β-haemolytic) & *E. coli*, *Streptococcus* sp. (β-haemolytic) & *Streptococcus* sp. (α-haemolytic), and *E. coli* & *Streptococcus* sp. (α-haemolytic) were the most recurrent bacterial combination for both sampling methods, independently of the inflammatory status. These findings align with previous reports describing similar microbial profiles in mares, in which *Streptococcus* sp. (β-haemolytic), particularly *S. zooepidemicus*, and *E. coli* have been frequently isolated from the equine uterus, either alone or in combination with other bacteria [5,17–19]. However, to the authors’ knowledge, this is the first large-scale study to integrate bacterial culture data with cytological findings for both US and UL samples.

Within both sampling techniques, the link between bacterial growth and uterine inflammation followed a similar profile. In US samples, the probability of EI was substantially greater in culture-positive samples than in culture-negative samples. However, among positively isolated samples, the number of bacterial isolates in US did not influence EI probability. This absence of an isolate-dependent effect likely reflects limited statistical power due to the low prevalence of EI and the small number of positive samples within each isolate category. Considering the bacterial species, the presence of *Streptococcus* sp. (β-haemolytic), *E. coli*, or *Streptococcus* sp. (α-haemolytic) was associated with increased risk of EI but, when stratifying by the number of isolates, only *Streptococcus* sp. (β-haemolytic) had a significant effect in US samples, in agreement with previous research [15]. Nevertheless, the low overall prevalence of EI in the population (4.8%) in US samples limited the statistical power to detect additional species-level or any co-occurrence associations with disease status. In addition, sampling limitations inherent to swabbing may have resulted in inadequate bacterial harvesting or determination of the inflammatory status due to incomplete endometrial coverage. To mitigate this concern, only samples containing endometrial epithelial cells in the cytology slide were included in the statistical analysis (as evaluated by a specialized technician), as their presence indicates adequate contact with the endometrial surface and supports reliability of both cytological and bacteriological findings. It should be noted that previous studies using US proposed monocultures as likely pathogenic isolates compared to mixed bacterial growth, being associated with impaired reproductive performance, including clinical endometritis and fertility issues [15,16,20]. In contrast to these findings, the number of isolates in our dataset did not alter the predicted probability of EI. This discrepancy might be due to differences in the prevalence of positive bacterial cultures and EI, as well as the target population (using swabs specifically for diagnosis of suspected cases) and the reproductive outcome studied (i.e., EI vs pregnancy outcome or viability of the foal).

Both the bacterial culture outcome and the number of isolates influenced EI probability in UL samples. The likelihood of EI was greater in samples with positive compared to negative culture, and also in samples with a single isolate compared with those with ≥3 isolates. This pattern suggests that single-isolate infections may represent true pathogenic infection, whereas samples with multiple bacterial species more likely reflect transient contamination or the presence of background (commensal) microbiota that does not induce an inflammatory response. The healthy equine uterus is not sterile [21], and infection is characterized by persistent mucosal inflammation and the presence of dysbiosis characterized by dominance of virulent pathogens [22]. Although one of the main advantages of UL is that it recovers both cells and microorganisms from a broader area of the endometrium, the probability of sample contamination is greater compared to US [3], particularly if the procedure was not performed under appropriate hygiene conditions or with deficient technique. Furthermore, some bacteria recovered from UL may represent normal residents of the microbiota of healthy mares, including *Streptococcus* sp., and *Staphylococcus* sp., and their presence should be interpreted cautiously, especially in cases of mixed growth [22]. Therefore, the evaluation of the species isolated in combination with cytology and clinical findings is essential when determining the appropriate treatment following UL sampling.

*Streptococcus* sp. (β-haemolytic) was associated with greater predicted probability of EI in US and UL samples. This finding is consistent with the well-stablished role of *Streptococcus* sp. (β-haemolytic), especially *Streptococcus equi* subspecies *zooepidemicus*, as major uterine pathogens in mares. Indeed, the latter is frequently isolated from EI-positive cases, being reported in up to 54% of culture-positive samples in this species [23,24]. Nevertheless, it is also part of the normal bacterial flora of the caudal reproductive tract of mares [25] and the main hypothesis is that this bacterium causes infectious endometritis by ascending through the genital tract, surpassing the anatomical barriers [26]. Therefore, the establishment of the disease depends on factors related to the uterine defence mechanisms, as well as specific, more specialized endometrial pathogenic strains [25]. It should be noted that, when *Streptococcus* sp. (β-haemolytic) was isolated in combination with *E. coli*, the prevalence of EI was lesser compared to samples in which this pair was absent. Together with the greater predicted probability of EI observed when isolated individually, these findings suggest that truly pathogenic *Streptococcus* sp. (β-haemolytic) strains may be capable of inducing a dysbiotic uterine environment, becoming the predominant microorganism and displacing components of the physiological flora. Moreover, it is also hypothesised that equine *S. zooepidemicus* may have the capacity to form biofilms, similar to that of *S. zooepidemicus* of porcine origin [27], which would enhance its ability to evade host defences and reduce antimicrobial efficacy.

Only in UL samples, the individual isolation of *S. aureus* was associated with increased probability of EI. This bacterium has been described as a constituent of the microbiota of the mare genital tract, residing mainly in the vaginal vestibule and clitoral fossa, and is considered as an opportunistic pathogen, although contamination during sampling cannot be entirely excluded [28]. In this regard, previous studies have linked this species to fewer positive cytological findings compared to *S. zooepidemicus* or *Klebsiella* [20,22]. Nevertheless, positive endometrial cytology or abnormal vaginal discharge, have also been documented in mares in which *S. aureus* was isolated, indicating that this microorganism can also be involved in true uterine infection [28,29]. Additionally, *S. aureus* expresses multiple virulence factors that promote adhesion, biofilm formation, and tissue damage [30]. In this context, although no data on mare endometritis are available in the literature, studies in other species provide relevant insights. Notably, Zhao et al. [31] reported that 96.2% of *S. aureus* strains isolated from cows with clinical endometritis in China harboured a large and diverse distribution of superantigen genes, with the *selj* gen being the most prevalent. These findings suggest that superantigen-related virulence factors may play an important role in the development or persistence of the condition. The variable findings across studies and the lack of information about virulent strains in mares highlight the need for further research using standardized sampling and analytical methods to clarify the pathogenic role of S. aureus in equine endometritis.

The presence of *E. coli* in US samples, and of *E. coli* or *Streptococcus* sp. (α-haemolytic) in UL samples was associated with lesser predicted probability of EI. Although *E. coli* has been described as a potential cause of endometritis, strain-level virulence varies widely, and many isolates recovered from the mare’s reproductive tract may represent transient colonizers rather than primary pathogens [21]. Additionally, previous research has described that *E. coli* is less likely to be associated with cytological findings than *Streptococcus* sp. (β-haemolytic) [16], although this minor inflammatory response does not imply absence of infection. In this way, the pathogenic potential of *E. coli* is closely linked to its ability to adhere to the host endometrial tissue, form a biofilm, and exhibit resistance to multiple classes of antimicrobials [32]. Of particular interest is the *fimH* gene, a major virulence determinant that has been detected in all *E. coli* strains recovered from mares with suspected bacterial endometritis, either alone or in combination with other virulence markers [32]. Similar results have been reported in cattle, where intrauterine *E. coli* strains carrying the virulence factor gene *fimH* were the most prevalent, suggesting its potential as predictor of metritis and endometritis in that species [33]. In the present study, strain-level data for *E. coli* were not available. Consequently, no possible differentiation between the strains present in EI-positive and EI-negative mares was assessed. Therefore, further research is needed to determine whether specific *E. coli* strains or particular virulence profiles are associated with endometritis in mares.

*Streptococcus* sp. (α-haemolytic) are generally regarded as commensal or low-virulence organisms in the reproductive tract of mares, being associated with low grade or no inflammation on cytological examination [15]. Previous work similarly reported that *Streptococcus* sp. (α-haemolytic) were more frequently detected as part of mixed bacterial growth rather than in pure culture (27% vs. 13%, respectively; [24]). In the present study, these species appeared as single isolates in only 2% for US and 1% for UL samples, whereas it was part of mixed cultures in 6% and 13% of cases, respectively. These findings support the hypothesis that certain bacteria recovered by culture may represent background microbiota, particularly within the UL dataset, and that their presence should not automatically prompt antimicrobial treatment in the absence of cytological evidence of inflammation.

The lack of association observed between most bacterial co-occurrence patterns and EI in UL suggests that many co-occurrences represent transient colonisation or background microbiota rather than synergistic pathogenicity. In addition, culture-based methods capture only part of the uterine microbial ecosystem and may underestimate fastidious or non-culturable organisms, obscuring true biological interactions [21]. In the recent years, culture-independent methods, such as 16S rRNA amplicon sequencing, have grown in popularity to characterize the reproductive tract microbiota in mares, as well as to identify shifts in its composition depending on health status [34–36]. Future culture-independent studies will be important to clarify how microbial community structures contribute to uterine inflammation.

The findings of this study have important implications for antimicrobial stewardship in equine reproductive practice. First, both cytology and bacteriological results should be considered to assess if the mare has endometritis and to clarify if the disease has an infectious origin. Then, appropriate antimicrobial therapy should be chosen accounting for the pathogenic bacterial species isolated and its antibiogram. It is necessary to consider that mixed bacterial growth, especially when involving low-virulence or commensal organisms, may not warrant antimicrobial therapy in the absence of cytological evidence of disease. Additionally, the World Health Organization made public a list of bacteria resistant to the action of specific antimicrobial drugs. Among the bacteria listed, *S. aureus* was included in the high-burden resistant pathogens on the 2024 WHO Bacterial Priority Pathogens List [37]. In our study, this bacterium was associated with increased predicted probability of EI, which urges antimicrobial susceptibility testing to guide drug selection, and also prompts the search for alternative or adjunctive therapies, including antibiofilm agents, non-steroidal anti-inflammatory drugs, chemical curettage, regenerative medicine, and probiotics [38–42]. Adopting such a targeted, bacterial-specific and cytology-guided approach may reduce unnecessary antimicrobial exposure, preserve treatment efficacy, and contribute to broader efforts to mitigate AMR in veterinary and human health.

This is the first large-scale study to integrate cytology and bacteriology results obtained from both US and UL datasets. This work provides valuable population level insight into the prevalence of EI, patterns of bacterial isolation, and species-specific associations with uterine inflammation. Importantly, our findings underscore key limitations in relying on culture alone to diagnose endometritis and highlight the importance of combining cytology with pathogen identity to avoid unnecessary antimicrobial use. Finally, several knowledge gaps identified should be addressed in future research, including pathogenic potential of specific bacterial species, strain-level virulence factors, and the role of uterine microbiota in health and disease. Altogether, this will improve diagnostic accuracy, promote responsible antimicrobial use, and enhance reproductive performance in broodmares.

## Conclusion

Positive bacterial growth was associated with increased likelihood of EI in both sampling techniques. In UL samples, cultures yielding a single bacterial isolate exhibited greater predicted probability of EI than cultures with ≥3 isolates. *Streptococcus* sp. (β -haemolytic) was associated with greater predicted probability of EI, whereas *E. coli* and *Streptococcus* sp. (α-haemolytic) were more frequently associated with lesser EI probability. These findings underscore the importance of interpreting bacterial culture results in conjunction with cytology and pathogen identity, rather than culture alone. This integrative approach can help differentiate true infection from contamination, guide targeted therapy, and support antimicrobial stewardship in broodmare reproductive management.

## Supporting information

S1 Table Frequency of uterine swabs (US) and low-volume uterine lavage (UL) collected from mares in Ireland classified according to their cytological evaluation of endometrial inflammation (EI). Endometrial inflammation was scored semi-quantitatively based on polymorphonuclear neutrophil (PMN) occurrence in at least three randomly selected high-power fields (HPF) at 40 × . Samples scored as 1 + , 2 + , 3 + , 4 + were considered positive for EI, with 1 + defined as 2 PMNs/HPF. Increasing scores indicate greater numbers of PMNs and a higher degree of inflammation.
(DOCX)

S2 Table Frequency of uterine swabs (US) and low-volume uterine lavage (UL) collected from mares in Ireland classified according to their cytological evaluation of endometrial inflammation (EI) and the number of isolates observed after bacterial culture. Endometrial inflammation was scored semi-quantitatively based on polymorphonuclear neutrophil (PMN) occurrence in at least three randomly selected high-power fields (HPF) at 40 × . Samples scored as 1 + , 2 + , 3 + , 4 + were considered positive for EI, with 1 + defined as 2 PMNs/HPF. Increasing scores indicate greater numbers of PMNs and a higher degree of inflammation.
(DOCX)

S3 Table Distribution of bacterial combinations identified in uterine swab samples from mares with and without endometrial inflammation (EI). Samples were considered positive to EI if ≥2 polymorphonuclear neutrophils per high-power field were observed, and negative otherwise.
(DOCX)

S4 Table Associations between most prevalent (>1%) bacterial species and endometrial inflammation (EI) in uterine swabs from mares, determined by generalized mixed-effects models. Samples were considered positive to EI if ≥2 polymorphonuclear neutrophils per high-power field were observed, and negative otherwise.
(DOCX)

S5 Table Distribution of bacterial combinations identified in low-volume uterine lavage samples from mares with and without endometrial inflammation (EI). Only bacterial species with prevalence >1% were included. Only bacterial pairs with more than 10 counts per group and valid Fisher’s P-values were retained for display. Samples were considered positive to EI if ≥2 polymorphonuclear neutrophils per high-power field were observed, and negative otherwise.
(DOCX)

S6 Table Associations between most prevalent (>1%) bacterial species and endometrial inflammation (EI) in low-volume uterine lavages from mares, determined by generalized mixed-effects models. Samples were considered positive to EI if ≥2 polymorphonuclear neutrophils per high-power field were observed, and negative otherwise.
(DOCX)
