# Supplementary material for: Evidence-based interpretation of uterine cultures in mares: Linking bacterial growth patterns to endometrial inflammation
Source: PLoS One. 2026 Aug 25;21(8):e0356822. doi: 10.1371/journal.pone.0356822 (PMC13505902; doi:10.1371/journal.pone.0356822)
Supplement: Supplementary file 1 [file pone.0356822.s007.pdf]

The manuscript **Evidence-based interpretation of uterine cultures in mares: linking bacterial growth patterns to cytological endometriti**s represents a large-scale study, clear and practical for veterinary clinicians

Despite the simple diagnostic methods used, the elevate number of samples contribute to the practical impact of the study, being relevant for clinical studies in equine reproduction

I recommend paper to be publish we some additional information, namely related to the scoring process for cytological evaluation (%); culture grow interpretation (contamination) e estrous phase (estrous versus diestrus). Other data concerning clinical signs were not available but it was explained in the discussion. If it is not possible it is recommended to be published as case report. It will be important to present data concerning bacterial resistance to antibiotics.

Line 84 it must be clarified it should be defined the number of bacterial above which we suspect of contamination
Line 87 the objectives must be more "objective" relating the results of the culture, cytology and technique used to collect the samples
Line 104 it is not possible to know the estrous phase? it is different do compare mares in estrous and diestrus
Line 115 the percentage of PMN will be more objective as the number will be affected by the collection technique. No other data was assessed? Cellular debris for instance?

Line 222 was the quadratic effect of number of isolates evaluated? If as discussed, it can happen that for more than 3 isolates it can be contamination it could be related to the quadratic effect. It is a usual practice in microbiology labs to consider more than 3 isolates as contamination

Line 229 it is not clear how the predicted probability is greater for more than 3 isolates compared to 1 isolate, concerning table 1 and it is not in consonance with results of UL in line 261-264
