# Supplementary material for: Evidence-based interpretation of uterine cultures in mares: Linking bacterial growth patterns to endometrial inflammation
Source: PLoS One. 2026 Aug 25;21(8):e0356822. doi: 10.1371/journal.pone.0356822 (PMC13505902; doi:10.1371/journal.pone.0356822)
Supplement: Supplementary file 2 [file pone.0356822.s001.docx]

**S1 Table**: Frequency of uterine swabs (US) and low-volume uterine lavage (UL) collected from mares in Ireland classified according to their cytological evaluation of endometrial inflammation (EI). Endometrial inflammation was scored semi-quantitatively based on polymorphonuclear neutrophil (PMN) occurrence in at least three randomly selected high-power fields (HPF) at 40×. Samples scored as 1+, 2+, 3+, 4+ were considered positive for EI, with 1+ defined as 2 PMNs/HPF. Increasing scores indicate greater numbers of PMNs and a higher degree of inflammation.

| **Sample type** | **Negative** | **Occasional** | **1+** | **2+** | **3+** | **4+** |
| --- | --- | --- | --- | --- | --- | --- |
| **US** | 1299 | 165 | 50 | 18 | 9 | 4 |
| **UL** | 1021 | 244 | 207 | 200 | 192 | 202 |
