# Supplementary material for: Evidence-based interpretation of uterine cultures in mares: Linking bacterial growth patterns to endometrial inflammation
Source: PLoS One. 2026 Aug 25;21(8):e0356822. doi: 10.1371/journal.pone.0356822 (PMC13505902; doi:10.1371/journal.pone.0356822)
Supplement: Supplementary file 3 [file pone.0356822.s002.docx]

**S2 Table:** Frequency of uterine swabs (US) and low-volume uterine lavage (UL) collected from mares in Ireland classified according to their cytological evaluation of endometrial inflammation (EI) and the number of isolates observed after bacterial culture. Endometrial inflammation was scored semi-quantitatively based on polymorphonuclear neutrophil (PMN) occurrence in at least three randomly selected high-power fields (HPF) at 40×. Samples scored as 1+, 2+, 3+, 4+ were considered positive for EI, with 1+ defined as 2 PMNs/HPF. Increasing scores indicate greater numbers of PMNs and a higher degree of inflammation.

| **Sample type** | **Number of isolates** | **Endometrial inflammation** | | | | | |
| --- | --- | --- | --- | --- | --- | --- | --- |
|  |  | **Negative** | **Occasional** | **1+** | **2+** | **3+** | **4+** |
| **US** | 0 | 743 | 51 | 6 | 3 | 0 | 0 |
|  | 1 | 323 | 61 | 23 | 7 | 4 | 3 |
|  | 2 | 184 | 36 | 13 | 6 | 3 | 0 |
|  | ≥3 | 49 | 17 | 8 | 2 | 2 | 1 |
| **UL** | 0 | 265 | 36 | 19 | 8 | 9 | 11 |
|  | 1 | 316 | 70 | 92 | 91 | 84 | 99 |
|  | 2 | 278 | 80 | 56 | 66 | 77 | 64 |
|  | ≥3 | 162 | 58 | 40 | 35 | 22 | 28 |
