# Supplementary material for: Evidence-based interpretation of uterine cultures in mares: Linking bacterial growth patterns to endometrial inflammation
Source: PLoS One. 2026 Aug 25;21(8):e0356822. doi: 10.1371/journal.pone.0356822 (PMC13505902; doi:10.1371/journal.pone.0356822)
Supplement: Supplementary file 4 [file pone.0356822.s003.docx]

**S3 Table.** Distribution of bacterial combinations identified in uterine swab samples from mares with and without endometrial inflammation (EI). Samples were considered positive to EI if ≥2 polymorphonuclear neutrophils per high-power field were observed, and negative otherwise.

| **Bacterial combination** | **Endometrial inflammation** | |
| --- | --- | --- |
|  | **No (%)** | **Yes (%)** |
| *Streptococcus* sp. (β-haemolytic) & *Escherichia coli* | 138 (89.0) | 17 (11.0) |
| *Streptococcus* sp. (α-haemolytic) & *Streptococcus* sp. (β-haemolytic) | 52 (86.7) | 8 (13.3) |
| *Streptococcus* sp. (α-haemolytic) & *Escherichia coli* | 35 (83.3) | 7 (16.7) |
| *Streptococcus* sp*. (*β-haemolytic*)* & *Staphylococcus aureus* | 28 (87.5) | 4 (12.5) |
| *Streptococcus* sp*. (*β-haemolytic*)* & *Staphylococcus* sp*.* | 23 (85.2) | 4 (14.8) |
| *Escherichia coli* & *Staphylococcus aureus* | 14 (87.5) | 2 (12.5) |
| *Streptococcus* sp. (α-haemolytic) & *Staphylococcus* sp. | 11 (84.6) | 2 (15.4) |
| *Streptococcus sp. (*β-haemolytic) & *Klebsiella aerogenes* | 8 (80.0) | 2 (20.0) |
| *Escherichia coli* & *Klebsiella aerogenes* | 7 (77.8) | 2 (22.2) |
| *Escherichia coli* & *Staphylococcus* sp*.* | 6 (75.0) | 2 (25.0) |

Percentages are based on the total number of samples per row.
