# Supplementary material for: Evidence-based interpretation of uterine cultures in mares: Linking bacterial growth patterns to endometrial inflammation
Source: PLoS One. 2026 Aug 25;21(8):e0356822. doi: 10.1371/journal.pone.0356822 (PMC13505902; doi:10.1371/journal.pone.0356822)
Supplement: Supplementary file 5 [file pone.0356822.s004.docx]

**S4 Table**: Associations between most prevalent (>1%) bacterial species and endometrial inflammation (EI) in uterine swabs from mares, determined by generalized mixed-effects models. Samples were considered positive to EI if ≥2 polymorphonuclear neutrophils per high-power field were observed, and negative otherwise.

| **Bacterium** | **Species detected** | **Predicted probability (± SE) of EI (%)** | **P-value (Tukey)** |
| --- | --- | --- | --- |
| *Streptococcus* sp. (β-haemolytic) | No (n = 21) | 2.0 ± 0.4 | <0.001 |
|  | Yes (n = 60) | 11.6 ± 1.4 |  |
| *Staphylococcus aureus* | No (n = 76) | 5.1 ± 0.6 | 0.09 |
|  | Yes (n = 5) | 10.8 ± 4.6 |  |
| *Escherichia coli* | No (n = 57) | 4.5 ± 0.5 | 0.01 |
|  | Yes (n = 24) | 8.3 ± 1.6 |  |
| *Klebsiella aerogenes* | No (n = 77) | 5.1 ± 0.6 | 0.01 |
|  | Yes (n = 4) | 18.1 ± 8.3 |  |
| *Staphylococcus* sp*.* | No (n = 76) | 5.1 ± 0.6 | 0.21 |
|  | Yes (n = 5) | 9.0 ± 3.9 |  |
| *Streptococcus* sp*. (*α-haemolytic) | No (n = 69) | 4.9 ± 0.6 | 0.02 |
|  | Yes (n = 12) | 9.7 ± 2.7 |  |

SE: standard error.
