# Supplementary material for: Evidence-based interpretation of uterine cultures in mares: Linking bacterial growth patterns to endometrial inflammation
Source: PLoS One. 2026 Aug 25;21(8):e0356822. doi: 10.1371/journal.pone.0356822 (PMC13505902; doi:10.1371/journal.pone.0356822)
Supplement: Supplementary file 6 [file pone.0356822.s005.docx]

**S5 Table.** Distribution of bacterial combinations identified in low-volume uterine lavage samples from mares with and without endometrial inflammation (EI). Only bacterial species with prevalence >1% were included. Only bacterial pairs with more than 10 counts per group and valid Fisher’s P-values were retained for display. Samples were considered positive to EI if ≥2 polymorphonuclear neutrophils per high-power field were observed, and negative otherwise.

| **Bacterial combination** | **Endometrial inflammation** | | ***P*-value** |
| --- | --- | --- | --- |
|  | **No (%)** | **Yes (%)** |  |
| *Streptococcus* sp. (β-haemolytic) & *Escherichia coli* | 307 (57.1) | 231 (42.9) | 0.02 |
| *Streptococcus* sp. (α-haemolytic) & *Streptococcus* sp. (β-haemolytic) | 119 (63.3) | 69 (36.7) | 0.58 |
| *Streptococcus* sp. (α-haemolytic) & *Escherichia coli* | 122 (65.6) | 64 (34.4) | 0.21 |
| *Streptococcus* sp*. (*β-haemolytic*)* & *Staphylococcus* sp*.* | 69 (63.9) | 39 (36.1) | 0.61 |
| *Escherichia coli* & *Staphylococcus* sp. | 62 (66.7) | 31 (33.3) | 0.33 |
| *Streptococcus* sp*. (*β-haemolytic*)* & *Staphylococcus aureus* | 42 (52.5) | 38 (47.5) | 0.13 |
| *Streptococcus* sp. (α-haemolytic) & *Staphylococcus* sp*.* | 47 (81.0) | 11 (19.0) | 0.001 |
| *Escherichia coli* & *Staphylococcus aureus* | 30 (57.7) | 22 (42.3) | 0.66 |
| *Streptococcus sp. (*β-haemolytic) & *Klebsiella aerogenes* | 27 (57.4) | 20 (42.6) | 0.65 |
| *Escherichia coli* & *Klebsiella aerogenes* | 31 (68.9) | 14 (31.1) | 0.35 |
| *Streptococcus sp. (*β-haemolytic) & *Corynebacterium* sp. | 20 (60.6) | 13 (39.4) | 0.99 |
| *Escherichia coli* & *Klebsiella pneumoniae* ssp. *pneumoniae* | 18 (64.3) | 10 (35.7) | 0.85 |
| *Streptococcus sp. (*β-haemolytic) & *Klebsiella pneumoniae* ssp. *pneumoniae* | 12 (46.2) | 14 (53.8) | 0.16 |

Percentages are based on the total number of samples per row.
