# Supplementary material for: Evidence-based interpretation of uterine cultures in mares: Linking bacterial growth patterns to endometrial inflammation
Source: PLoS One. 2026 Aug 25;21(8):e0356822. doi: 10.1371/journal.pone.0356822 (PMC13505902; doi:10.1371/journal.pone.0356822)
Supplement: Supplementary file 7 [file pone.0356822.s006.docx]

**S6 Table**: Associations between most prevalent (>1%) bacterial species and endometrial inflammation (EI) in low-volume uterine lavages from mares, determined by generalized mixed-effects models. Samples were considered positive to EI if ≥2 polymorphonuclear neutrophils per high-power field were observed, and negative otherwise.

| **Bacterium** | **Species detected** | **Predicted probability (± SE) of EI (%)** | **P-value (Tukey)** |
| --- | --- | --- | --- |
| *Streptococcus* sp. (β-haemolytic) | No (n = 204) | 23.4 ± 1.9 | <0.001 |
|  | Yes (n = 597) | 48.1 ± 2.1 |  |
| *Staphylococcus aureus* | No (n = 734) | 36.5 ± 1.9 | 0.001 |
|  | Yes (n = 67) | 51.2 ± 4.9 |  |
| *Escherichia coli* | No (n = 469) | 36.4 ± 2.3 | 0.37 |
|  | Yes (n = 332) | 38.3 ± 2.2 |  |
| *Klebsiella pneumoniae* ssp*. pneumoniae* | No (n = 781) | 37.3 ± 2.0 | 0.27 |
|  | Yes (n = 20) | 45.8 ± 8.0 |  |
| *Klebsiella aerogenes* | No (n = 773) | 37.5 ± 2.0 | 0.73 |
|  | Yes (n = 28) | 35.5 ± 5.8 |  |
| *Staphylococcus* sp*.* | No (n = 742) | 37.9 ± 2.0 | 0.17 |
|  | Yes (n = 59) | 32.5 ± 3.9 |  |
| *Streptococcus* sp*. (*α-haemolytic) | No (n = 702) | 38.6 ± 2.1 | 0.01 |
|  | Yes (n = 99) | 30.6 ± 3.2 |  |
| *Corynebacterium* sp. | No (n = 786) | 37.6 ± 2.0 | 0.32 |
|  | Yes (n = 15) | 30.4 ± 7.0 |  |

SE: standard error.
