# Supplementary material for: Evidence-based interpretation of uterine cultures in mares: Linking bacterial growth patterns to endometrial inflammation
Source: PLoS One. 2026 Aug 25;21(8):e0356822. doi: 10.1371/journal.pone.0356822 (PMC13505902; doi:10.1371/journal.pone.0356822)
Supplement: Supplementary file 8 [file pone.0356822.s008.odt]

Reviewers’ comments
Reviewer #1: 
Thank you for taking the time to submit your manscript for review. The manuscript has the potential to provide readers with some very useful and clinically applicable data. However as the manuscript currently stands, despite reading it many times it is very difficult to find any clarity, particularly in your results sections and tables which I find incoherent. The introduction and discussion bring up some important points but more clarifty in your aims, objectives and results would help.

We thank the reviewer for the thorough and constructive evaluation of our manuscript. We appreciate the concerns raised regarding the interpretation of cytological findings, the distinction between uterine inflammation and infectious endometritis, and the presentation of the results. In response to the comments, the manuscript has been revised to differentiate cytological evidence of endometrial inflammation from bacterial infection, avoid statements about diagnostic performance or causality, and clarify that US and UL samples were analysed independently throughout the study. The objectives were rephrased to improve clarity and the table presentation in the results was modified as suggested.

MAJOR ISSUES
Fundamentally cytology has repeatedly been proven to have a very poor se/sp for detecting bacterial endometritis. I believe this manuscript is over stating its importance and providing the reader with misinformation in its current state. Cytology is also not binary and simplifying the outcome to positive or negative is not providing enough insight. Fundamentally reporting that US samples having a positive cytology in 5.2% of samples and positive bacteria in 48% of samples highlights that cytology is not a very sensitive marker of bacterial endometritis contrary to what the manuscript states.
We agree that the relatively low prevalence of cytological inflammation compared with positive bacterial culture findings highlights that bacteriological isolation and cytological inflammation are not synonyms, and that cytology should not be considered a sensitive or specific test for the detection of bacterial infection. This observation reinforces the main message of the study, that positive bacterial culture results should not automatically be interpreted as evidence of clinically relevant uterine inflammation, and the use of antibiotics should be handled with care. To avoid confusion and improve the clarity of the manuscript, the text has been revised to emphasize that cytology was used as an indicator of uterine inflammatory status rather than as a diagnostic test for bacterial infection. The term cytological endometritis was changed to endometrial inflammation throughout the manuscript.

I have issues with the paper claiming that cytology and culture improves diagnostic accuracy as this simply was not tested in this paper. There is no discussion of sensitivity, specificity, NPV or PPV in this manuscript. For example it would appear from table 1 that mares with a negative cytology on UL was more likely to have a positive bacterial growth with 1 isolate (30.5%) than a negative bacterial growth (23.8%). I am not sure you can claim this is "evidence-based interpretation" in its current state, as descriptive data of laboratory submissions.
The main focus of this paper was to raise awareness on the fact that positive bacterial cultures do not always come with endometrial inflammation, and that the systematic use of antibiotics should be avoided unless inflammation is confirmed, not evaluate the usefulness of cytology to diagnosis bacterial endometritis. Based on the reviewer’s comment, this focus was not clear enough in the first version of the manuscript. Therefore, the term “diagnostic accuracy” was changed to “interpretation of bacteriological findings” to avoid confusion. Additionally, we would like to mention that results from Table 1 are merely descriptive, with no statistical tests applied. Descriptive data cannot be directly compared with the results derived from the model, as the latter includes more variables into consideration as the year and the farm. Table 1 was included to give a general picture of the data obtained for each sampling technique.

The cytology grading system needs clarifying i.e. how many PMN’s is associated with each grade not just “higher number indicating higher severity of condition”. I imagine there is a significant difference between 1+ and 4+ and this needs to be clarified, cytology is not binary.
Unfortunately, the retrospective laboratory database did not provide the exact number of PMN associated with each cytological category. Cytological slides were evaluated according to the laboratory’s routine diagnostic procedures, in which inflammatory severity was recorded using the ordinal scoring system mentioned in the manuscript. We have clarified this in the Materials and Methods section (L119-125) and also acknowledge in the discussion (L339-343) that this scoring system could have introduced some bias regarding UL samples.
We agree that a difference exists between scores 1+ and 4+ regarding the number of PMN. However, the objective of the present study was not to investigate the severity of endometrial inflammation, but rather the association between bacteriological findings and the presence of cytological evidence of inflammation. Consequently, cytology was analysed as a binary outcome (positive or negative) based on the laboratory’s diagnostic classification.

Also what condition are you diagnosing with cytology? Definition of cytological endometritis needs clarifying, infact i would refer to it as uterine inflammation as that is what you are reporting. Endometrial inflammation (measured by PMNs) can occur in the uterus in response to many things other than infectious endometritis, such as pneumovagina, urovagina, mucous, semen and during uterine involution post foaling. Without any clinical information regarding these mares such as when the swabs were taken or if the mares were presenting with other clinical signs it is very important to clarify this. Again stating that “streptococcus as the primary determinant of endometrial inflammation” cannot be stated with the data available just that is was the bacteria most associated with evidence of uterine inflammation.
The former term “cytological endometritis” (now replaced by endometrial inflammation”), is defined as an increased proportion of polymorphonuclear cells (PMN) in endometrial cytology. We agree that the number of PMN can be increased in other situations rather than endometritis, as the reviewer pointed out. For this reason, the manuscript has been revised throughout to emphasize that cytology was used as an indicator of endometrial inflammation rather than as a direct measure of bacterial infection. We acknowledge that the absence of individual mare-level information limits the ability to determine the cause of inflammation in individual cases. However, the objective of the study was to investigate associations between bacteriological findings and cytological evidence of inflammation at the population level rather than establish causality in individual mares. To avoid confusion on this matter, the term “clinical” (L348) was changed to “individual”. The sentence “…as the primary determinant of endometrial inflammation” was changed to “primary bacteria associated with evidence of endometrial inflammation” (L31).

Sampling the endometrium via an endometrial swab and a low volume lavage are completely different methods and whilst this is commented on in the discussion it would appear despite the declarations they are in fact comparing the two. The act of a uterine lavage is often done to help recruit healthy” neutrophils by transient irritation so maybe more PMNs may be expected in this method? Without as you say performing both methods on the same mare at the same time how can you compare. Also as stated it is possible that samples for lavage were on mares presenting with clinical signs of bacterial endometritis or infertility and that the swabs were routine pre-breeding? I am wondering if it would be better to completely seperate the two. Also how do we know without any fertility data the significance of the bacteria and PMNs. Perhaps an endometrial lavage is more sensitive for detecting the normal uterine microbiome and hence more bacteria? How do we know if this is pathogenic and needs antimicrobial treatment or not? Or were they just contaminants? I think you need to be careful in overstating the use of cytology to guide decision making on antimicrobial use without any clinical information.
We agree that US and UL samples represent distinct sampling techniques and that direct comparison between them would require paired sampling from the same mare, as acknowledge in the discussion. However, direct comparison between techniques was not an objective of the present study. US and UL samples were analysed independently throughout the study, and separate generalized mixed-effects models were fitted for each sampling method. Consequently, all reported associations between bacteriological and cytological findings were derived within each sampling technique. Although differences in the prevalence of endometrial inflammation and bacterial isolation were described, these observations were not subjected to direct statistical comparison and should not be interpreted as evidence of greater accuracy of one technique above the other. We added a small discussion on this matter because we considered it an interesting finding, and give possible reasons for that difference. Comparison between the effectiveness of each technique to diagnose endometritis in mares was never within the scope of the study, as our dataset did not allow it. Some clarifications were added in the discussion to avoid further confusion on this matter (L326, L361-362)

No indication as the population of mares tested? What breed? What age? What method was used for collecting the samples. Were the swabs double guarded or not?
In the database provided by the laboratory, no individual mare-level information about the breed or age was available. This is clarified in the manuscript (L105-106) and acknowledge as a limitation in the discussion (L348-350). Because sample collection was performed under routine field conditions by multiple practitioners (the veterinarian was tested as random effect in the models), detailed information regarding the sampling devices used (single or double-guarded swabs) was not consistently recorded in the laboratory database. Although not included in the manuscript, we would like to clarify that the laboratory highly encourages practitioners to use double-guarded swabs.

Culture plates were assessed for”profusion and purity of bacteria” yet no further mention of profusion mentioned. A single colony of bacteria is very different from a profuse growth. The amount of bacterial growth should be included in the manuscript. Table 2 would be more useful to quantify the amount of growth of bacteria rather than the number of isolates.Also the overall bacterial growth is much higher than in other papers, an important point which doesn't seem to be discussed?
Unfortunately, although culture plates were routinely evaluated for purity and profusion during laboratory processing, quantitative growth-density data were not consistently retained in the retrospective database available for analysis. Consequently, these variables could not be incorporated into the statistical models. We removed this information from the materials and methods.

All the tables need addressing and the number of each sample added in each column to understand the significance. Table 2 makes little sense how can you have streptococcus, 1,2,3 number of isolates (assuming this means bacteria in addition to BHS but no idea what bacteria) and then the next column saying bacterial isolated no and yes???? Is this referring to PMNs on cytology?? Again without knowing how many swabs in each category I have no idea if any of this information is relevant. Also no idea how they are deciding that streptococcus is the main bacteria on the plate how many colonies of BHS, what other bacteria and how many colonies grown???
I am struggling to tell from the manuscript how many samples had a pure growth of BHS, E.coli etc etc and which had a mixed growth?
Again table 3 how many samples had these combinations? I would put these tables in supplementary material.
The total number of samples for each category is included in all tables. Considering Table 2, it reflects the results for the generalized mixed-effects models fitted for US. For each bacterium, the model considered the number of isolates in all samples, and if the bacterium was isolated or not in each category of isolates. The sum for each isolate category in the different bacteria is the same, so including the number of samples in each column would be redundant and we prefer to include the number in the third column, as we consider it the most informative one. This associations were only evaluated for the most prevalent bacterial species (>1%), as indicated in the Table caption. 
Inclusion of bacterial species as a fixed effect did not imply that it was the predominant isolate on the plate. Rather, it indicated if that species was present or absent in the sample, either alone or in combination with other bacterial species. No information about the number of colonies for each bacterial species was provided. As currently design, the present study evaluated the presence/absence of the bacteria and its link to endometrial inflammation, alone or in combination, independent of the number of colonies/bacterial load.
The number of samples that had single or mixed growth for each bacterial species in both US and UL is included in Tables 2 and 4. This tables include the information for each bacterium according to the number of isolates detected in each sample.
For Table 3, the number of samples that had each combination of bacteria is the sum of the columns No and Yes of endometrial inflammation. According to the reviewer’s suggestions, this table was moved to the Supplementary materials.

Endometrial biopsies are often described as “gold standard” diagnosis for endometritis this should be discussed especially in the introduction where it claims that swabs may not reflect the overall uterine health status but in contrast a UL may? This is also not true as both are only sampling the surface of the uterus.
The clarification that biopsy is the gold standard for the diagnosis of endometritis is included in the introduction as suggested (L63-65). The drawbacks of endometrial diagnosis are also described, justifying why this technique is not used unless specifically required in field conditions due to its invasive nature and additional laboratory processing required compared to lavages or swabs.
We agree that both US and UL assess only the luminal environment and do not provide information regarding deeper endometrial architecture. Our intention was not to suggest that UL reflects overall uterine health status to the same extent as biopsy. Want we meant is that, because UL samples recover material from a larger proportion of endometrial surface, they may increase the likelihood of detecting localized luminal inflammation compared with a focal swab sample.

E.coli is often reported not to be associated with an inflammatory response, yet this is not mentioned at all. Suggesting “low pathogenicity” as it was associated with lesser CE, is potentially dangerous especially since CE is known to be very poor for detecting endometritis but E,coli is proven to be a predictor for poor live foaling outcomes.
In the discussion it is described that “previous research has described that E. coli is less likely to be associated with cytological findings than Streptococcus sp. (β-haemolytic) [16], although this minor inflammatory response does not imply absence of infection” (L448-450). Additionally, this phenomena is further discussed in L451-455).

SUGGESTIONS
I suggest the authors concentrate the paper and particularly the tables of results to provide a more succinct manuscript.
According to the reviewer’s suggestions, the Results section and tables were revised to improve clarity and readability. To improve the presentation of the findings, tables containing supporting information were moved to the Supplementary Materials, while the main manuscript now retains only those tables considered essential for understanding the most important results. In addition, new supplementary tables were included to provide greater detail regarding bacterial isolation patterns and cytological findings as suggested, without interrupting the flow of the main text. We believe these revisions have resulted in a more concise and accessible presentation of the data while preserving the information necessary for the interpretation of the study findings.

1/ Frequency of different bacterial species cultured from swabs and lavages including amount of growth, concentrating initally on those samples with a pure growth of a single species (this is not clear).
This information is provided in Tables 2 and 3 in the manuscript. According to the reviewer’s suggestions in the major comments about moving Table 3 to supplementary materials, only three tables should remain in the main manuscript, while a fourth table is shown in the supplementary material.

2/ Frequency of each cytology grade 0, occasional, 1+, 2+, 3+, 4+ for both the swab and lavage
The frequency of each cytology grade was included as S1 Table in the supplementary materials.

3/ Frequency of each cytology grade and its associating with a positive growth yes or no
4/ Frequency of each cytology grade and its associating with specific pure growth of bacteria.
The frequency of each cytology grade according to the number of isolated was included as S2 Table in the supplementary materials. This table includes the information required in the two comments above.

5/ The mixed growth associating with each cytology grade is interesting but the addition of 13 bacterial combinations in table 3 is incredibly confusing, I would add this to supplementary reading. I understand you want to highlight that BHS in any combination was associted with a greater likelihood of a positive cytology but this currently is lost.
Table 3 was moved to the supplementary materials as suggested.
Reviewer #2: 
The authors determined results from endometrial cytology and bacteriology in mares retrospectively. Results from a total of 1,545 samples from endometrial swabs and 2,066 from low volume uterine lavage were included. The data support the conclusion that the inclusion of results from endometrial cytology – even more when low volume uterine lavage is used – is beneficial regarding diagnostic accuracy. The finding is, however, not really new and in agreement with previous studies.

There are two major concerns regarding the experimental design of this study:
i) Results from samples collected as swab and as low volume uterine lavage are not directly comparable because they were not collected from the same mare.
ii) Information from the mare regarding i.e. her reproductive history, stage of cycle and any clinical information was not available because the laboratory could not disclose client-specific data.

This is a major setback regarding the reliability of results and conclusions. The authors address these limitations in the manuscript but that does not solve the problem. The manuscript is well written and especially the discussion is a pleasure to read. In contrast, the results section is not easy to follow and would benefit from improvement, also regarding its organisation. The manuscript deserves to be published but I am not convinced that this journal is the optimal choice. A journal with focus on animal reproduction could be better suited.
We thank the reviewer for the careful evaluation of the manuscript and for recognizing the practical relevance and the value of the large dataset, especially in Ireland, where no information regarding mare endometritis is currently available.
We acknowledge that the retrospective nature of the study and the absence of mare-level information represent important limitations. We have further emphasized and justify these limitations in the revised manuscript. Although oestrus cycle stage may have influenced the absolute prevalence of cytological positivity, we consider it unlikely to have altered the overall patterns of associations identified in this study. This is now clarified in the revised version in L350-357. Additionally, we believe that the large sample size provides valuable information regarding the association between bacteriological evidence of uterine inflammation under field conditions. Moreover, this study constitutes the first large-scale epidemiological characterization of bacteriological and cytological findings in Irish mares, generating population-level data under routine field conditions that were previously unavailable. 
Regarding the concern that US and UL samples were not collected from the same mare, we agree that this precludes direct comparison between sampling techniques. However, this was never the objective of the present study. Instead, each sampling method was analysed independently using separate statistical models, and all conclusions regarding bacterial growth patterns and species-specific associations were derived within each sampling technique. Consequently, although the lack of paired sampling limits comparisons between techniques, it does not affect the validity of the results. This is clarified in the discussion (L361-362).
We appreciate the reviewer’ suggestion regarding journal scope. Although PLOS One is not a scientific journal focused on equine medicine or animal reproduction, it welcomes original research submissions from the natural sciences. Despite agreeing with the reviewer’s point of view, we also think that our work falls into the scope and remains appropriate for the readership of this journal. Additionally, the open access nature of the journal may facilitate the dissemination of the findings to both researchers and  practitioners working in equine veterinary medicine and reproduction, maximizing their potential impact.

The following points should be addressed:
How can you guarantee that the techniques used by the many veterinarians involved sample collection were comparable?
Information about the veterinarian who performed the sample collection was provided (coded) by the laboratory and included in the database. In the statistical analysis, the variable veterinarian was tested as a random effect in the generalized mixed-effects model for both US and UL. However, the inclusion in the model did not improve its fit and was therefore removed. While this does not eliminate procedural variability, it indicates that veterinarian-specific effects did not explain a substantial proportion of the variation within the dataset.

What was the basis to decide whether an endometrial swab or a low volume uterine lavage was used for sample collection? Was this case-specific or a veterinarian-specific decision?
The veterinarian who performed the sampling was the one who made the decision. We worked with the database provided by the laboratory that processed the samples sent by the veterinarians. Based on laboratory experience over the years, veterinarians usually collect US for screening before breeding and UL when they suspect uterine pathology. However, because this information was anecdotal and unavailable for individual mares, it was not included in the statistical analysis. 

The farm where the mare was located was included as a random effect. This requires that more specific information on the farms is provided in the material and methods section.
We worked with the database provided by the laboratory that processed the samples. No information regarding farm size, management practices, or breeding systems was available, and the only data we can provide is that is that farms were located in Ireland (L95-96).

Why are p-values >0.05 given as exact values? Please provise a reasonable explanation or change to not significant.
P-values > 0.05 are reported as > lowest p-value obtained in the group of data involved, not exact values. For example, in L232, the P > 0.17 indicates that there were no significant differences among the number of isolates, and that the lowest p-value obtained after post-hoc comparison was 0.17, being the p-values for the remaining comparisons always greater than 0.17.

Lines 505-512 should be deleted.
As those lines were a substantial part of the conclusion, and the conclusion is the final message the readers will remember, we think that a brief summary of the main results should remain. Therefore, we decided to summarize it. The new conclusion now reads like this: Positive bacterial growth was associated with increased likelihood of endometrial inflammation in both sampling techniques. In UL samples, cultures yielding a single bacterial isolate exhibited greater predicted probability of endometrial inflammation than cultures with ≥3 isolates.  Streptococcus sp. (β -haemolytic) was associated with greater predicted probability of endometrial inflammation, whereas E. coli and Streptococcus sp. (α-haemolytic) were more frequently associated with lesser inflammation probability. These findings underscore the importance of interpreting bacterial culture results in conjunction with cytology and pathogen identity, rather than culture alone. This integrative approach can help differentiate true infection from contamination, guide targeted therapy, and support antimicrobial stewardship in broodmare reproductive management (L514-523).

Reviewer #3: 
The manuscript Evidence-based interpretation of uterine cultures in mares: linking bacterial growth patterns to cytological endometritis represents a large-scale study, clear and practical for veterinary clinicians

Despite the simple diagnostic methods used, the elevate number of samples contribute to the practical impact of the study, being relevant for clinical studies in equine reproduction

I recommend paper to be publish we additional information, namely related to the scoring process for cytological evaluation (%); culture grow interpretation (contamination) e estrous phase (estrous versus diestrus). Other data concerning clinical signs were not available but it was explained in the discussion. If it is not possible it is recommended to be published as case report. It will be important to present data concerning bacterial resistance to antibiotics.
We thank the reviewer for the thorough and constructive evaluation of our manuscript. We agree that additional information regarding cytological scoring, interpretation of culture contamination, and the absence of oestrus cycle data required further clarification. Accordingly, the manuscript has been revised to clarify the consideration of the isolation of more than three bacterial species as potential contamination, and to further discuss the lack of information regarding reproductive stage as a limiting factor.
With regard to the scoring process for cytological evaluation, the protocol used by the laboratory was maintained. Although we agree that the PMN% would have been a better approach, this is a reference laboratory for equine samples in Ireland, and the same scoring system has also been used in previous research on mare endometritis. Therefore, it has been proved as a reliable technique for cytology evaluation and considering the number of samples processed annually by this laboratory, the results can be directly interpreted by equine practitioners.
Lastly, we agree that antimicrobial resistance is an important and clinically relevant topic. Unfortunately, antimicrobial susceptibility data were not available in this retrospective database and could not be evaluated in the present study. We have acknowledged in the discussion that these results have potential implications regarding antimicrobial resistance, and this manuscript should be considered as the start point of a bigger research project to improve the diagnosis of endometritis in order to promote antimicrobial stewardship.
Considering this information, we believe that the scale of the dataset, the analytical approach, and the novel information generated regarding bacterial growth patterns and species-specific associations are consistent with an original research study.
Line 84 it must be clarified it should be defined the number of bacterial above which we suspect of contamination
The manuscript was modified accordingly in L85-87. The following sentence was included in the hypothesis: … and that the isolation of more than three different bacterial species can be indicative of contamination.
Line 87 the objectives must be more “objective” relating the results of the culture, cytology and technique used to collect the samples
The objectives were modified accordingly. The original paragraph was replaced with “the objective of this large-scale retrospective study was to characterize the associations between single versus mixed bacterial growth and the prevalence of endometrial inflammation in samples collected using either US or UL. Additionally, we aimed to determine which bacterial species, alone or in combination, are most strongly linked to endometrial inflammation.” (L89-93).
Line 104 it is not possible to know the estrous phase? it is different do compare mares in estrous and diestrus
We agree with the reviewer that the stage of the oestrous cycle can influence endometrial cytology findings, and that interpretation of PMN counts may differ between oestrus and dioestrus. Unfortunately, information regarding reproductive stage was not available in the laboratory database and therefore could not be incorporated into the analysis. This limitation has been acknowledged in the discussion.
However, we believe that the absence of oestrous cycle information is unlikely to fully explain the observed associations. First, due to the large sample size and retrospective nature of the dataset, reproductive stage would be expected to act as a source of random biological variations rather than a systematic bias. This variation would be more likely to attenuate associations than to generate them. Second, out conclusions are based on the relationship between bacteriological findings and cytological evidence of inflammation. While mild neutrophil infiltration may occur physiologically during oestrus, this factor would not explain the consistent species-specific associations observed across a large number of samples. Consequently, although oestrus cycle stage may have influenced the absolute prevalence of cytological positivity, we consider it unlikely to have altered the overall patterns of associations identified in this study. This is now clarified in the revised version in L350-357.
Line 115 the percentage of PMN will be more objective as the number will be affected by the collection technique. No other data was assessed? Cellular debris for instance?
We agree with the reviewer that the use of PMN% may have provided a more objective assessment, and this was acknowledged in the discussion. Unfortunately, the laboratory database only contained the recorded PMN count category. Therefore, PMN% could not be determined retrospectively. To reduce the risk of including non-representative samples, only slides containing endometrial cells were retained for the analysis. The presence of epithelial cells was used as a quality-control criterion to confirm adequate contact with the endometrial surface for both sampling techniques. Although information regarding cellular debris was available, this parameter was not incorporated into the classification as it was not part of the diagnostic criteria.
Line 222 was the quadratic effect of number of isolates evaluated? If as discussed, it can happen that for more than 3 isolates it can be contamination it could be related to the quadratic effect. It is a usual practice in microbiology labs to consider more than 3 isolates as contamination
The number of isolates was analysed as a categorical variable rather than a continuous variable. Therefore, potential non-linear relationships were inherently evaluated through category-specific comparisons. Because isolate number was not treated as a continuous predictor, a quadratic term was not applicable.
Line 229 it is not clear how the predicted probability is greater for more than 3 isolates compared to 1 isolate, concerning table 1 and it is not in consonance with results of UL in line 261-264.
It should be noted that the results presented in Table 1 are merely descriptive, with no statistical tests performed. However, the numbers displayed in line 229 are the results for the generalized mixed-effects model built for uterine swabs. Descriptive data cannot be directly compared with the results derived from the model, as the latter includes more variables into consideration as the year and the farm. Table 1 was included to give a general picture of the data obtained for each sampling technique. 
As stated in the Materials and Methods, swabs and lavages must be treated independently, so separate models were built for each sampling technique. Thus, the results obtained from each model (L229 for US and L261-264 for UL) should also be interpreted independently from each other.
