# Supplementary material for: Evidence-based interpretation of uterine cultures in mares: Linking bacterial growth patterns to endometrial inflammation
Source: PLoS One. 2026 Aug 25;21(8):e0356822. doi: 10.1371/journal.pone.0356822 (PMC13505902; doi:10.1371/journal.pone.0356822)
Supplement: Peer review history

# Peer review: Decision Letter 0

**DOI:** 10.1371/journal.pone.0356822.r001

**Authors:** Dinesh Dadarwal

6 May 2026

Dear Dr. Yanez,

Thank you for submitting your manuscript to PLOS ONE. After careful consideration, we feel that it has merit but does not fully meet PLOS ONE’s publication criteria as it currently stands. Therefore, we invite you to submit a revised version of the manuscript that addresses the points raised during the review process.

Please submit your revised manuscript by Jun 20 2026 11:59PM. If you will need more time than this to complete your revisions, please reply to this message or contact the journal office at plosone@plos.org. When you're ready to submit your revision, log on to https://www.editorialmanager.com/pone/ and select the 'Submissions Needing Revision' folder to locate your manuscript file.

- A letter that responds to each point raised by the academic editor and reviewer(s). You should upload this letter as a separate file labeled 'Response to Reviewers'.
- A marked-up copy of your manuscript that highlights changes made to the original version. You should upload this as a separate file labeled 'Revised Manuscript with Track Changes'.
- An unmarked version of your revised paper without tracked changes. You should upload this as a separate file labeled 'Manuscript'.

If you would like to make changes to your financial disclosure, please include your updated statement in your cover letter. Guidelines for resubmitting your figure files are available below the reviewer comments at the end of this letter.

If applicable, we recommend that you deposit your laboratory protocols in protocols.io to enhance the reproducibility of your results. Protocols.io assigns your protocol its own identifier (DOI) so that it can be cited independently in the future. For instructions see: https://journals.plos.org/plosone/s/submission-guidelines#loc-laboratory-protocols. Additionally, PLOS ONE offers an option for publishing peer-reviewed Lab Protocol articles, which describe protocols hosted on protocols.io. Read more information on sharing protocols at https://plos.org/protocols?utm_medium=editorial-email&utm_source=authorletters&utm_campaign=protocols.

As the corresponding author, your ORCID iD is verified in the submission system and will appear in the published article. PLOS supports the use of ORCID, and we encourage all coauthors to register for an ORCID iD and use it as well. Please encourage your coauthors to verify their ORCID iD within the submission system before final acceptance, as unverified ORCID iDs will not appear in the published article. *Only*  the individual author can complete the verification step; PLOS staff *cannot*  verify ORCID iDs on behalf of authors.

We look forward to receiving your revised manuscript.

Kind regards,

Dinesh Dadarwal

Academic Editor

PLOS One

Journal Requirements:

When submitting your revision, we need you to address these additional requirements.

1. Please ensure that your manuscript meets PLOS ONE's style requirements, including those for file naming. The PLOS ONE style templates can be found at

https://journals.plos.org/plosone/s/file?id=wjVg/PLOSOne_formatting_sample_main_body.pdf and

https://journals.plos.org/plosone/s/file?id=ba62/PLOSOne_formatting_sample_title_authors_affiliations.pdf

2. Thank you for stating in your Funding Statement:

“This study was partly funded by the Equine Technical Support & Equine Breeding Scheme 2025 (project number: 9/ETS/2025) of the Department of Agriculture, Food and the Marine in Ireland. Uxía Yáñez holds a postdoctoral contract funded by the Xunta de Galicia (Ref. ED481B_033/2024), and Osvaldo Bogado Pascottini is supported by a UCD Ad Astra Fellowship.”

Please provide an amended statement that declares *all* the funding or sources of support (whether external or internal to your organization) received during this study, as detailed online in our guide for authors at http://journals.plos.org/plosone/s/submit-now. Please also include the statement “There was no additional external funding received for this study.” in your updated Funding Statement.

Please include your amended Funding Statement within your cover letter. We will change the online submission form on your behalf.

3. Thank you for stating the following financial disclosure:

“This study was partly funded by the Equine Technical Support & Equine Breeding Scheme 2025 (project number: 9/ETS/2025) of the Department of Agriculture, Food and the Marine in Ireland. Uxía Yáñez holds a postdoctoral contract funded by the Xunta de Galicia (Ref. ED481B_033/2024), and Osvaldo Bogado Pascottini is supported by a UCD Ad Astra Fellowship.”

Please state what role the funders took in the study. If the funders had no role, please state: "The funders had no role in study design, data collection and analysis, decision to publish, or preparation of the manuscript."

If this statement is not correct you must amend it as needed.

Please include this amended Role of Funder statement in your cover letter; we will change the online submission form on your behalf.

4. In the online submission form, you indicated that data is available by the corresponding author under reasonable request.

All PLOS journals now require all data underlying the findings described in their manuscript to be freely available to other researchers, either 1. In a public repository, 2. Within the manuscript itself, or 3. Uploaded as supplementary information.

This policy applies to all data except where public deposition would breach compliance with the protocol approved by your research ethics board. If your data cannot be made publicly available for ethical or legal reasons (e.g., public availability would compromise patient privacy), please explain your reasons on resubmission and your exemption request will be escalated for approval.

5.  Thank you for stating the following in the Competing Interests section:

“I have read the journal's policy and the authors of this manuscript have the following competing interests: James Gibbons was employed as a veterinary microbiologist at the Irish Equine Centre during the period of data collection. The remaining authors have declared that no competing interests exist.”

We note that one or more of the authors are employed by a commercial company: Waterside Equine Repro Services, Co and 3Irish Equine Centre, Johnstown, Naas, Co.

a. Please provide an amended Funding Statement declaring this commercial affiliation, as well as a statement regarding the Role of Funders in your study. If the funding organization did not play a role in the study design, data collection and analysis, decision to publish, or preparation of the manuscript and only provided financial support in the form of authors' salaries and/or research materials, please review your statements relating to the author contributions, and ensure you have specifically and accurately indicated the role(s) that these authors had in your study. You can update author roles in the Author Contributions section of the online submission form.

Please also include the following statement within your amended Funding Statement.

“The funder provided support in the form of salaries for authors [insert relevant initials], but did not have any additional role in the study design, data collection and analysis, decision to publish, or preparation of the manuscript. The specific roles of these authors are articulated in the ‘author contributions’ section.”

If your commercial affiliation did play a role in your study, please state and explain this role within your updated Funding Statement.

b. Please also provide an updated Competing Interests Statement declaring this commercial affiliation along with any other relevant declarations relating to employment, consultancy, patents, products in development, or marketed products, etc.

Within your Competing Interests Statement, please confirm that this commercial affiliation does not alter your adherence to all PLOS ONE policies on sharing data and materials by including the following statement: "This does not alter our adherence to PLOS ONE policies on sharing data and materials.” (as detailed online in our guide for authors http://journals.plos.org/plosone/s/competing-interests) . If this adherence statement is not accurate and there are restrictions on sharing of data and/or materials, please state these. Please note that we cannot proceed with consideration of your article until this information has been declared.

Please include both an updated Funding Statement and Competing Interests Statement in your cover letter. We will change the online submission form on your behalf.

6. We note that Figure 1 in your submission contain copyrighted images. All PLOS content is published under the Creative Commons Attribution License (CC BY 4.0), which means that the manuscript, images, and Supporting Information files will be freely available online, and any third party is permitted to access, download, copy, distribute, and use these materials in any way, even commercially, with proper attribution. For more information, see our copyright guidelines: http://journals.plos.org/plosone/s/licenses-and-copyright.

We require you to either (1) present written permission from the copyright holder to publish these figures specifically under the CC BY 4.0 license, or (2) remove the figures from your submission:

a. You may seek permission from the original copyright holder of Figure 1 to publish the content specifically under the CC BY 4.0 license.

We recommend that you contact the original copyright holder with the Content Permission Form (http://journals.plos.org/plosone/s/file?id=7c09/content-permission-form.pdf) and the following text:

“I request permission for the open-access journal PLOS ONE to publish XXX under the Creative Commons Attribution License (CCAL) CC BY 4.0 (http://creativecommons.org/licenses/by/4.0/). Please be aware that this license allows unrestricted use and distribution, even commercially, by third parties. Please reply and provide explicit written permission to publish XXX under a CC BY license and complete the attached form.”

Please upload the completed Content Permission Form or other proof of granted permissions as an "Other" file with your submission.

In the figure caption of the copyrighted figure, please include the following text: “Reprinted from [ref] under a CC BY license, with permission from [name of publisher], original copyright [original copyright year].”

b. If you are unable to obtain permission from the original copyright holder to publish these figures under the CC BY 4.0 license or if the copyright holder’s requirements are incompatible with the CC BY 4.0 license, please either i) remove the figure or ii) supply a replacement figure that complies with the CC BY 4.0 license. Please check copyright information on all replacement figures and update the figure caption with source information. If applicable, please specify in the figure caption text when a figure is similar but not identical to the original image and is therefore for illustrative purposes only.

7.  Please include captions for your Supporting Information files at the end of your manuscript, and update any in-text citations to match accordingly. Please see our Supporting Information guidelines for more information: http://journals.plos.org/plosone/s/supporting-information.

8. If the reviewer comments include a recommendation to cite specific previously published works, please review and evaluate these publications to determine whether they are relevant and should be cited. There is no requirement to cite these works unless the editor has indicated otherwise.

Additional Editor Comments:

Thank you for submitting your manuscript to PLOS ONE for consideration. Based on evaluations from three independent reviewers, your manuscript requires substantial revisions before it can be considered suitable for publication. Please ensure that all comments and suggestions from each reviewer are addressed thoroughly and completely.

[Note: HTML markup is below. Please do not edit.]

Reviewers' comments:

Reviewer's Responses to Questions

**Comments to the Author**

1. Is the manuscript technically sound, and do the data support the conclusions?

Reviewer #1: No

Reviewer #2: Partly

Reviewer #3: Partly

**********

2. Has the statistical analysis been performed appropriately and rigorously?

Reviewer #1: No

Reviewer #2: Yes

Reviewer #3: Yes

**********

3. Have the authors made all data underlying the findings in their manuscript fully available?

The PLOS Data policy

Reviewer #1: Yes

Reviewer #2: Yes

Reviewer #3: Yes

**********

4. Is the manuscript presented in an intelligible fashion and written in standard English?

Reviewer #1: Yes

Reviewer #2: Yes

Reviewer #3: Yes

**********

Reviewer #1: Thank you for taking the time to submit your manscript for review. The manuscript has the potential to provide readers with some very useful and clinically applicable data. However as the manuscript currently stands, despite reading it many times it is very difficult to find any clarity, particularly in your results sections and tables which I find incoherent. The introduction and discussion bring up some important points but more clarifty in your aims, objectives and results would help.

MAJOR ISSUES

Fundamentally cytology has repeatedly been proven to have a very poor se/sp for detecting bacterial endometritis. I believe this manuscript is over stating its importance and providing the reader with misinformation in its current state. Cytology is also not binary and simplifying the outcome to positive or negative is not providing enough insight. Fundamentally reporting that US samples having a positive cytology in 5.2% of samples and positive bacteria in 48% of samples highlights that cytology is not a very sensitive marker of bacterial endometritis contrary to what the manuscript states.

I have issues with the paper claiming that cytology and culture improves diagnostic accuracy as this simply was not tested in this paper. There is no discussion of sensitivity, specificity, NPV or PPV in this manuscript. For example it would appear from table 1 that mares with a negative cytology on UL was more likely to have a positive bacterial growth with 1 isolate (30.5%) than a negative bacterial growth (23.8%). I am not sure you can claim this is "evidence-based interpretation" in its current state, as descriptive data of laboratory submissions.

The cytology grading system needs clarifying i.e. how many PMN’s is associated with each grade not just “higher number indicating higher severity of condition”. I imagine there is a significant difference between 1+ and 4+ and this needs to be clarified, cytology is not binary.

Also what condition are you diagnosing with cytology? Definition of cytological endometritis needs clarifying, infact i would refer to it as uterine inflammation as that is what you are reporting. Endometrial inflammation (measured by PMNs) can occur in the uterus in response to many things other than infectious endometritis, such as pneumovagina, urovagina, mucous, semen and during uterine involution post foaling. Without any clinical information regarding these mares such as when the swabs were taken or if the mares were presenting with other clinical signs it is very important to clarify this. Again stating that “streptococcus as the primary determinant of endometrial inflammation” cannot be stated with the data available just that is was the bacteria most associated with evidence of uterine inflammation.

Sampling the endometrium via an endometrial swab and a low volume lavage are completely different methods and whilst this is commented on in the discussion it would appear despite the declarations they are in fact comparing the two. The act of a uterine lavage is often done to help recruit healthy” neutrophils by transient irritation so maybe more PMNs may be expected in this method? Without as you say performing both methods on the same mare at the same time how can you compare. Also as stated it is possible that samples for lavage were on mares presenting with clinical signs of bacterial endometritis or infertility and that the swabs were routine pre-breeding? I am wondering if it would be better to completely seperate the two. Also how do we know without any fertility data the significance of the bacteria and PMNs. Perhaps an endometrial lavage is more sensitive for detecting the normal uterine microbiome and hence more bacteria? How do we know if this is pathogenic and needs antimicrobial treatment or not? Or were they just contaminants? I think you need to be careful in overstating the use of cytology to guide decision making on antimicrobial use without any clinical information.

No indication as the population of mares tested? What breed? What age? What method was used for collecting the samples. Were the swabs double guarded or not?

Culture plates were assessed for”profusion and purity of bacteria” yet no further mention of profusion mentioned. A single colony of bacteria is very different from a profuse growth. The amount of bacterial growth should be included in the manuscript. Table 2 would be more useful to quantify the amount of growth of bacteria rather than the number of isolates.Also the overall bacterial growth is much higher than in other papers, an important point which doesn't seem to be discussed?

All the tables need addressing and the number of each sample added in each column to understand the significance. Table 2 makes little sense how can you have streptococcus, 1,2,3 number of isolates (assuming this means bacteria in addition to BHS but no idea what bacteria) and then the next column saying bacterial isolated no and yes???? Is this referring to PMNs on cytology?? Again without knowing how many swabs in each category I have no idea if any of this information is relevant. Also no idea how they are deciding that streptococcus is the main bacteria on the plate how many colonies of BHS, what other bacteria and how many colonies grown???

I am struggling to tell from the manuscript how many samples had a pure growth of BHS, E.coli etc etc and which had a mixed growth?

Again table 3 how many samples had these combinations? I would put these tables in supplementary material.

Endometrial biopsies are often described as “gold standard” diagnosis for endometritis this should be discussed especially in the introduction where it claims that swabs may not reflect the overall uterine health status but in contrast a UL may? This is also not true as both are only sampling the surface of the uterus.

E.coli is often reported not to be associated with an inflammatory response, yet this is not mentioned at all. Suggesting “low pathogenicity” as it was associated with lesser CE, is potentially dangerous especially since CE is known to be very poor for detecting endometritis but E,coli is proven to be a predictor for poor live foaling outcomes.

SUGGESTIONS

I suggest the authors concentrate the paper and particularly the tables of results to provide a more succinct manuscript.

1/ Frequency of different bacterial species cultured from swabs and lavages including amount of growth, concentrating initally on those samples with a pure growth of a single species (this is not clear).

2/ Frequency of each cytology grade 0, occasional, 1+, 2+, 3+, 4+ for both the swab and lavage

3/ Frequency of each cytology grade and its associating with a positive growth yes or no

4/ Frequency of each cytology grade and its associating with specific pure growth of bacteria.

5/ The mixed growth associating with each cytology grade is interesting but the addition of 13 bacterial combinations in table 3 is incredibly confusing, I would add this to supplementary reading. I understand you want to highlight that BHS in any combination was associted with a greater likelihood of a positive cytology but this currently is lost.

Reviewer #2: The authors determined results from endometrial cytology and bacteriology in mares retrospectively. Results from a total of 1,545 samples from endometrial swabs and 2,066 from low volume uterine lavage were included. The data support the conclusion that the inclusion of results from endometrial cytology – even more when low volume uterine lavage is used – is beneficial regarding diagnostic accuracy. The finding is, however, not really new and in agreement with previous studies.

There are two major concerns regarding the experimental design of this study:

i) Results from samples collected as swab and as low volume uterine lavage are not directly comparable because they were not collected from the same mare.

ii) Information from the mare regarding i.e. her reproductive history, stage of cycle and any clinical information was not available because the laboratory could not disclose client-specific data.

This is a major setback regarding the reliability of results and conclusions. The authors address these limitations in the manuscript but that does not solve the problem. The manuscript is well written and especially the discussion is a pleasure to read. In contrast, the results section is not easy to follow and would benefit from improvement, also regarding its organisation. The manuscript deserves to be published but I am not convinced that this journal is the optimal choice. A journal with focus on animal reproduction could be better suited.

The following points should be addressed:

How can you guarantee that the techniques used by the many veterinarians involved sample collection were comparable?

What was the basis to decide whether an endometrial swab or a low volume uterine lavage was used for sample collection? Was this case-specific or a veterinarian-specific decision?

The farm where the mare was located was included as a random effect. This requires that more specific information on the farms is provided in the material and methods section.

Why are p-values >0.05 given as exact values? Please provise a reasonable explanation or change to not significant.

Lines 505-512 should be deleted.

Reviewer #3: The manuscript Evidence-based interpretation of uterine cultures in mares: linking bacterial growth patterns to cytological endometritis represents a large-scale study, clear and practical for veterinary clinicians

Despite the simple diagnostic methods used, the elevate number of samples contribute to the practical impact of the study, being relevant for clinical studies in equine reproduction

I recommend paper to be publish we additional information, namely related to the scoring process for cytological evaluation (%); culture grow interpretation (contamination) e estrous phase (estrous versus diestrus). Other data concerning clinical signs were not available but it was explained in the discussion. If it is not possible it is recommended to be published as case report. It will be important to present data concerning bacterial resistance to antibiotics.

**********

what does this mean?). If published, this will include your full peer review and any attached files.

If you choose “no”, your identity will remain anonymous but your review may still be made public.

**Do you want your identity to be public for this peer review?**  For information about this choice, including consent withdrawal, please see our Privacy Policy

Reviewer #1: No

Reviewer #2: No

Reviewer #3: No

**********

[NOTE: If reviewer comments were submitted as an attachment file, they will be attached to this email and accessible via the submission site. Please log into your account, locate the manuscript record, and check for the action link "View Attachments". If this link does not appear, there are no attachment files.]

To ensure your figures meet our technical requirements, please review our figure guidelines: https://journals.plos.org/plosone/s/figures

You may also use PLOS’s free figure tool, NAAS, to help you prepare publication quality figures: https://journals.plos.org/plosone/s/figures#loc-tools-for-figure-preparation.

NAAS will assess whether your figures meet our technical requirements by comparing each figure against our figure specifications.

Attachment Submitted filename: recomendations.pdf

# Author comment: Author response to Decision Letter 1

**DOI:** 10.1371/journal.pone.0356822.r002

18 Jun 2026

Reviewers’ comments

Reviewer #1:

Thank you for taking the time to submit your manscript for review. The manuscript has the potential to provide readers with some very useful and clinically applicable data. However as the manuscript currently stands, despite reading it many times it is very difficult to find any clarity, particularly in your results sections and tables which I find incoherent. The introduction and discussion bring up some important points but more clarifty in your aims, objectives and results would help.

We thank the reviewer for the thorough and constructive evaluation of our manuscript. We appreciate the concerns raised regarding the interpretation of cytological findings, the distinction between uterine inflammation and infectious endometritis, and the presentation of the results. In response to the comments, the manuscript has been revised to differentiate cytological evidence of endometrial inflammation from bacterial infection, avoid statements about diagnostic performance or causality, and clarify that US and UL samples were analysed independently throughout the study. The objectives were rephrased to improve clarity and the table presentation in the results was modified as suggested.

MAJOR ISSUES

Fundamentally cytology has repeatedly been proven to have a very poor se/sp for detecting bacterial endometritis. I believe this manuscript is over stating its importance and providing the reader with misinformation in its current state. Cytology is also not binary and simplifying the outcome to positive or negative is not providing enough insight. Fundamentally reporting that US samples having a positive cytology in 5.2% of samples and positive bacteria in 48% of samples highlights that cytology is not a very sensitive marker of bacterial endometritis contrary to what the manuscript states.

We agree that the relatively low prevalence of cytological inflammation compared with positive bacterial culture findings highlights that bacteriological isolation and cytological inflammation are not synonyms, and that cytology should not be considered a sensitive or specific test for the detection of bacterial infection. This observation reinforces the main message of the study, that positive bacterial culture results should not automatically be interpreted as evidence of clinically relevant uterine inflammation, and the use of antibiotics should be handled with care. To avoid confusion and improve the clarity of the manuscript, the text has been revised to emphasize that cytology was used as an indicator of uterine inflammatory status rather than as a diagnostic test for bacterial infection. The term cytological endometritis was changed to endometrial inflammation throughout the manuscript.

I have issues with the paper claiming that cytology and culture improves diagnostic accuracy as this simply was not tested in this paper. There is no discussion of sensitivity, specificity, NPV or PPV in this manuscript. For example it would appear from table 1 that mares with a negative cytology on UL was more likely to have a positive bacterial growth with 1 isolate (30.5%) than a negative bacterial growth (23.8%). I am not sure you can claim this is "evidence-based interpretation" in its current state, as descriptive data of laboratory submissions.

The main focus of this paper was to raise awareness on the fact that positive bacterial cultures do not always come with endometrial inflammation, and that the systematic use of antibiotics should be avoided unless inflammation is confirmed, not evaluate the usefulness of cytology to diagnosis bacterial endometritis. Based on the reviewer’s comment, this focus was not clear enough in the first version of the manuscript. Therefore, the term “diagnostic accuracy” was changed to “interpretation of bacteriological findings” to avoid confusion. Additionally, we would like to mention that results from Table 1 are merely descriptive, with no statistical tests applied. Descriptive data cannot be directly compared with the results derived from the model, as the latter includes more variables into consideration as the year and the farm. Table 1 was included to give a general picture of the data obtained for each sampling technique.

The cytology grading system needs clarifying i.e. how many PMN’s is associated with each grade not just “higher number indicating higher severity of condition”. I imagine there is a significant difference between 1+ and 4+ and this needs to be clarified, cytology is not binary.

Unfortunately, the retrospective laboratory database did not provide the exact number of PMN associated with each cytological category. Cytological slides were evaluated according to the laboratory’s routine diagnostic procedures, in which inflammatory severity was recorded using the ordinal scoring system mentioned in the manuscript. We have clarified this in the Materials and Methods section (L119-125) and also acknowledge in the discussion (L339-343) that this scoring system could have introduced some bias regarding UL samples.

We agree that a difference exists between scores 1+ and 4+ regarding the number of PMN. However, the objective of the present study was not to investigate the severity of endometrial inflammation, but rather the association between bacteriological findings and the presence of cytological evidence of inflammation. Consequently, cytology was analysed as a binary outcome (positive or negative) based on the laboratory’s diagnostic classification.

Also what condition are you diagnosing with cytology? Definition of cytological endometritis needs clarifying, infact i would refer to it as uterine inflammation as that is what you are reporting. Endometrial inflammation (measured by PMNs) can occur in the uterus in response to many things other than infectious endometritis, such as pneumovagina, urovagina, mucous, semen and during uterine involution post foaling. Without any clinical information regarding these mares such as when the swabs were taken or if the mares were presenting with other clinical signs it is very important to clarify this. Again stating that “streptococcus as the primary determinant of endometrial inflammation” cannot be stated with the data available just that is was the bacteria most associated with evidence of uterine inflammation.

The former term “cytological endometritis” (now replaced by endometrial inflammation”), is defined as an increased proportion of polymorphonuclear cells (PMN) in endometrial cytology. We agree that the number of PMN can be increased in other situations rather than endometritis, as the reviewer pointed out. For this reason, the manuscript has been revised throughout to emphasize that cytology was used as an indicator of endometrial inflammation rather than as a direct measure of bacterial infection. We acknowledge that the absence of individual mare-level information limits the ability to determine the cause of inflammation in individual cases. However, the objective of the study was to investigate associations between bacteriological findings and cytological evidence of inflammation at the population level rather than establish causality in individual mares. To avoid confusion on this matter, the term “clinical” (L348) was changed to “individual”. The sentence “…as the primary determinant of endometrial inflammation” was changed to “primary bacteria associated with evidence of endometrial inflammation” (L31).

Sampling the endometrium via an endometrial swab and a low volume lavage are completely different methods and whilst this is commented on in the discussion it would appear despite the declarations they are in fact comparing the two. The act of a uterine lavage is often done to help recruit healthy” neutrophils by transient irritation so maybe more PMNs may be expected in this method? Without as you say performing both methods on the same mare at the same time how can you compare. Also as stated it is possible that samples for lavage were on mares presenting with clinical signs of bacterial endometritis or infertility and that the swabs were routine pre-breeding? I am wondering if it would be better to completely seperate the two. Also how do we know without any fertility data the significance of the bacteria and PMNs. Perhaps an endometrial lavage is more sensitive for detecting the normal uterine microbiome and hence more bacteria? How do we know if this is pathogenic and needs antimicrobial treatment or not? Or were they just contaminants? I think you need to be careful in overstating the use of cytology to guide decision making on antimicrobial use without any clinical information.

We agree that US and UL samples represent distinct sampling techniques and that direct comparison between them would require paired sampling from the same mare, as acknowledge in the discussion. However, direct comparison between techniques was not an objective of the present study. US and UL samples were analysed independently throughout the study, and separate generalized mixed-effects models were fitted for each sampling method. Consequently, all reported associations between bacteriological and cytological findings were derived within each sampling technique. Although differences in the prevalence of endometrial inflammation and bacterial isolation were described, these observations were not subjected to direct statistical comparison and should not be interpreted as evidence of greater accuracy of one technique above the other. We added a small discussion on this matter because we considered it an interesting finding, and give possible reasons for that difference. Comparison between the effectiveness of each technique to diagnose endometritis in mares was never within the scope of the study, as our dataset did not allow it. Some clarifications were added in the discussion to avoid further confusion on this matter (L326, L361-362)

No indication as the population of mares tested? What breed? What age? What method was used for collecting the samples. Were the swabs double guarded or not?

In the database provided by the laboratory, no individual mare-level information about the breed or age was available. This is clarified in the manuscript (L105-106) and acknowledge as a limitation in the discussion (L348-350). Because sample collection was performed under routine field conditions by multiple practitioners (the veterinarian was tested as random effect in the models), detailed information regarding the sampling devices used (single or double-guarded swabs) was not consistently recorded in the laboratory database. Although not included in the manuscript, we would like to clarify that the laboratory highly encourages practitioners to use double-guarded swabs.

Culture plates were assessed for”profusion and purity of bacteria” yet no further mention of profusion mentioned. A single colony of bacteria is very different from a profuse growth. The amount of bacterial growth should be included in the manuscript. Table 2 would be more useful to quantify the amount of growth of bacteria rather than the number of isolates.Also the overall bacterial growth is much higher than in other papers, an important point which doesn't seem to be discussed?

Unfortunately, although culture plates were routinely evaluated for purity and profusion during laboratory processing, quantitative growth-density data were not consistently retained in the retrospective database available for analysis. Consequently, these variables could not be incorporated into the statistical models. We removed this information from the materials and methods.

All the tables need addressing and the number of each sample added in each column to understand the significance. Table 2 makes little sense how can you have streptococcus, 1,2,3 number of isolates (assuming this means bacteria in addition to BHS but no idea what bacteria) and then the next column saying bacterial isolated no and yes???? Is this referring to PMNs on cytology?? Again without knowing how many swabs in each category I have no idea if any of this information is relevant. Also no idea how they are deciding that streptococcus is the main bacteria on the plate how many colonies of BHS, what other bacteria and how many colonies grown???

I am struggling to tell from the manuscript how many samples had a pure growth of BHS, E.coli etc etc and which had a mixed growth?

Again table 3 how many samples had these combinations? I would put these tables in supplementary material.

The total number of samples for each category is included in all tables. Considering Table 2, it reflects the results for the generalized mixed-effects models fitted for US. For each bacterium, the model considered the number of isolates in all samples, and if the bacterium was isolated or not in each category of isolates. The sum for each isolate category in the different bacteria is the same, so including the number of samples in each column would be redundant and we prefer to include the number in the third column, as we consider it the most informative one. This associations were only evaluated for the most prevalent bacterial species (>1%), as indicated in the Table caption.

Inclusion of bacterial species as a fixed effect did not imply that it was the predominant isolate on the plate. Rather, it indicated if that species was present or absent in the sample, either alone or in combination with other bacterial species. No information about the number of colonies for each bacterial species was provided. As currently design, the present study evaluated the presence/absence of the bacteria and its link to endometrial inflammation, alone or in combination, independent of the number of colonies/bacterial load.

The number of samples that had single or mixed growth for each bacterial species in both US and UL is included in Tables 2 and 4. This tables include the information for each bacterium according to the number of isolates detected in each sample.

For Table 3, the number of samples that had each combination of bacteria is the sum of the columns No and Yes of endometrial inflammation. According to the reviewer’s suggestions, this table was moved to the Supplementary materials.

Endometrial biopsies are often described as “gold standard” diagnosis for endometritis this should be discussed especially in the introduction where it claims that swabs may not reflect the overall uterine health status but in contrast a UL may? This is also not true as both are only sampling the surface of the uterus.

The clarification that biopsy is the gold standard for the diagnosis of endometritis is included in the introduction as suggested (L63-65). The drawbacks of endometrial diagnosis are also described, justifying why this technique is not used unless specifically required in field conditions due to its invasive nature and additional laboratory processing required compared to lavages or swabs.

We agree that both US and UL assess only the luminal environment and do not provide information regarding deeper endometrial architecture. Our intention was not to suggest that UL reflects overall uterine health status to the same extent as biopsy. Want we meant is that, because UL samples recover material from a larger proportion of endometrial surface, they may increase the likelihood of detecting localized luminal inflammation compared with a focal swab sample.

E.coli is often reported not to be associated with an inflammatory response, yet this is not mentioned at all. Suggesting “low pathogenicity” as it was associated with lesser CE, is potentially dangerous especially since CE is known to be very poor for detecting endometritis but E,coli is proven to be a predictor for poor live foaling outcomes.

In the discussion it is described that “previous research has described that E. coli is less likely to be associated with cytological findings than Streptococcus sp. (β-haemolytic) [16], although this minor inflammatory response does not imply absence of infection” (L448-450). Additionally, this phenomena is further discussed in L451-455).

SUGGESTIONS

I suggest the authors concentrate the paper and particularly the tables of results to provide a more succinct manuscript.

According to the reviewer’s suggestions, the Results section and tables were revised to improve clar

Attachment Submitted filename: Response to Reviewers.odt

# Peer review: Decision Letter 1

**DOI:** 10.1371/journal.pone.0356822.r003

**Authors:** Dinesh Dadarwal

5 Aug 2026

Dear Dr. Yanez,

Thank you for submitting your manuscript to PLOS ONE. After careful consideration, we feel that it has merit but does not fully meet PLOS ONE’s publication criteria as it currently stands. Therefore, we invite you to submit a revised version of the manuscript that addresses the points raised during the review process.

Please submit your revised manuscript by Sep 19 2026 11:59PM. If you will need more time than this to complete your revisions, please reply to this message or contact the journal office at plosone@plos.org. When you're ready to submit your revision, log on to https://www.editorialmanager.com/pone/ and select the 'Submissions Needing Revision' folder to locate your manuscript file.

- A letter that responds to each point raised by the academic editor and reviewer(s). You should upload this letter as a separate file labeled 'Response to Reviewers'.
- A marked-up copy of your manuscript that highlights changes made to the original version. You should upload this as a separate file labeled 'Revised Manuscript with Track Changes'.
- An unmarked version of your revised paper without tracked changes. You should upload this as a separate file labeled 'Manuscript'.

If you would like to make changes to your financial disclosure, please include your updated statement in your cover letter. Guidelines for resubmitting your figure files are available below the reviewer comments at the end of this letter.

If applicable, we recommend that you deposit your laboratory protocols in protocols.io to enhance the reproducibility of your results. Protocols.io assigns your protocol its own identifier (DOI) so that it can be cited independently in the future. For instructions see: https://journals.plos.org/plosone/s/submission-guidelines#loc-laboratory-protocols. Additionally, PLOS ONE offers an option for publishing peer-reviewed Lab Protocol articles, which describe protocols hosted on protocols.io. Read more information on sharing protocols at https://plos.org/protocols?utm_medium=editorial-email&utm_source=authorletters&utm_campaign=protocols.

As the corresponding author, your ORCID iD is verified in the submission system and will appear in the published article. PLOS supports the use of ORCID, and we encourage all coauthors to register for an ORCID iD and use it as well. Please encourage your coauthors to verify their ORCID iD within the submission system before final acceptance, as unverified ORCID iDs will not appear in the published article. *Only*  the individual author can complete the verification step; PLOS staff *cannot*  verify ORCID iDs on behalf of authors.

We look forward to receiving your revised manuscript.

Kind regards,

Dinesh Dadarwal

Academic Editor

PLOS One

Journal Requirements:

If the reviewer comments include a recommendation to cite specific previously published works, please review and evaluate these publications to determine whether they are relevant and should be cited. There is no requirement to cite these works unless the editor has indicated otherwise.

Please review your reference list to ensure that it is complete and correct. If you have cited papers that have been retracted, please include the rationale for doing so in the manuscript text, or remove these references and replace them with relevant current references. Any changes to the reference list should be mentioned in the rebuttal letter that accompanies your revised manuscript. If you need to cite a retracted article, indicate the article’s retracted status in the References list and also include a citation and full reference for the retraction notice.

Additional Editor Comments:

Thank you for submitting your revised manuscript. The main concerns from the first round have been adequately resolved, and the remaining points are ones inherent to the dataset (unpaired sampling, no individual mare-level data) rather than issues a further rewrite could fix. Your claims are now appropriately hedged to match what the data support, so I am recommending minor revision.

Please address before resubmission:

Your response letter attributes new text to L339–343 (centrifugation-related bias in UL cytology). This passage closely resembles wording already present in the original submission — please verify and correct the response letter accordingly.

Do a final pass of the Discussion to remove any wording suggesting UL is more diagnostically reliable than US, since the manuscript states the two techniques were assessed independently and are not directly comparable.

Confirm consistent use of "endometrial inflammation (EI)" throughout the Results, Discussion, figures, tables, and Supporting Information — no leftover instances of "cytological endometritis" or "CE."

Check that table and citation numbering are still correct after moving the former Table 3 to Supporting Information.

[Note: HTML markup is below. Please do not edit.]

Reviewers' comments:

Reviewer's Responses to Questions

**Comments to the Author**

Reviewer #1: (No Response)

Reviewer #2: (No Response)

**********

2. Is the manuscript technically sound, and do the data support the conclusions?

Reviewer #1: Yes

Reviewer #2: Partly

**********

3. Has the statistical analysis been performed appropriately and rigorously?

Reviewer #1: I Don't Know

Reviewer #2: Yes

**********

4. Have the authors made all data underlying the findings in their manuscript fully available?

The PLOS Data policy

Reviewer #1: Yes

Reviewer #2: Yes

**********

5. Is the manuscript presented in an intelligible fashion and written in standard English?

Reviewer #1: Yes

Reviewer #2: Yes

**********

Reviewer #1: Thank you for taking the time to address my previous concerns.

Minor comments

Adding Fig 2/3/4 into the main manuscript may help clarify the descriptive results for readers.

I can’t find anywhere a mention that in table 1 more mares with negative EI on UL had positive bacterial growth (30.5%) compared to those with no bacteria (23.8%). If you are including this table you should clarify this point. Line 313/314 seems to contradict this.

Table 2 is still confusing and I feel it could be represented in a clearer way.

Line 49: Consequently BACTERIAL/INFECTIONS endometritis diagnostic accuracy…

Line 55: Add reference for double guarded being less prone to contamination

Line 105: Study could have been conducted at the farm level so worth stating this in limitations of the study

Line 342: Proportion of PMNs to epithelial cells may be more sensitive/specific

Reviewer #2: The manuscript has been throughly reviewed by 3 independent reviewers who all raised very serious concerns regarding the experimental protocol and the data analysis. I cannot say that I am impressed how the authors handled this revision. The reviewers' concerns have mainly been rebutted but there are no considerable changes in the manuscript. This reviewer is also still not convinced that the manuscript really fits into the scope of this journal.

**********

what does this mean?). If published, this will include your full peer review and any attached files.

If you choose “no”, your identity will remain anonymous but your review may still be made public.

**Do you want your identity to be public for this peer review?**  For information about this choice, including consent withdrawal, please see our Privacy Policy

Reviewer #1: No

Reviewer #2: No

**********

[NOTE: If reviewer comments were submitted as an attachment file, they will be attached to this email and accessible via the submission site. Please log into your account, locate the manuscript record, and check for the action link "View Attachments". If this link does not appear, there are no attachment files.]

To ensure your figures meet our technical requirements, please review our figure guidelines: https://journals.plos.org/plosone/s/figures

You may also use PLOS’s free figure tool, NAAS, to help you prepare publication quality figures: https://journals.plos.org/plosone/s/figures#loc-tools-for-figure-preparation.

NAAS will assess whether your figures meet our technical requirements by comparing each figure against our figure specifications.

# Author comment: Author response to Decision Letter 2

**DOI:** 10.1371/journal.pone.0356822.r004

6 Aug 2026

The authors would like to thank the editor and the reviewers for taking their time to revise the manuscript a second time and make the necessary comments and suggestions to improve both its clarity and quality. Below are our detailed responses to each comment.

Editor’s comments

Thank you for submitting your revised manuscript. The main concerns from the first round have been adequately resolved, and the remaining points are ones inherent to the dataset (unpaired sampling, no individual mare-level data) rather than issues a further rewrite could fix. Your claims are now appropriately hedged to match what the data support, so I am recommending minor revision.

The authors would like to thank the editor for the constructive feedback given in this review round. The manuscript has been modified according to the suggestions and a detailed response to each of the comments is provided below.

Please address before resubmission:

Your response letter attributes new text to L339–343 (centrifugation-related bias in UL cytology). This passage closely resembles wording already present in the original submission — please verify and correct the response letter accordingly.

The authors would like to clarify their response from the previous review round. To address the reviewer’s comment, we added new text to the Materials and Methods section to further explain the cytology grading system used following the laboratory’s routine evaluation protocols. The original version already included a paragraph acknowledging the potential bias associated with applying the same PMN threshold to both sampling methods (former L339-343). In our previous response, we intended only to indicate that this limitation had already been acknowledge in the Discussion, not that new information was added. That is why the wording remained essentially unchanged, except for minor nomenclature modifications. We apologize for the confusing wording in our previous response. The response letter has been modified to clearly distinguish between the new text added to the Materials and Methods section and the pre-existing discussion of this limitation.

The new answer now reads as follows:

Unfortunately, the retrospective laboratory database did not provide the exact number of PMN associated with each cytological category. Cytological slides were evaluated according to the laboratory’s routine diagnostic procedures, in which inflammatory severity was recorded using the ordinal scoring system mentioned in the manuscript. We have clarified this in the Materials and Methods section (L119-125). The discussion already acknowledged the potential limitations associated with applying the same PMN threshold to both sampling techniques (L339-343), such as the introduction of some bias regarding UL samples.

Do a final pass of the Discussion to remove any wording suggesting UL is more diagnostically reliable than US, since the manuscript states the two techniques were assessed independently and are not directly comparable.

The Discussion was revised to modify the wording suggesting direct comparison between sampling techniques. A clarification statement was added at the beginning of the Discussion (L317-322) and further modifications were made on L324, L326, L340-341, L344-L345, L385, L489, and L518.

Confirm consistent use of "endometrial inflammation (EI)" throughout the Results, Discussion, figures, tables, and Supporting Information — no leftover instances of "cytological endometritis" or "CE."

According to the editor’s suggestion, the consistent use of endometrial inflammation and its abbreviation (EI) has been carefully revised through the whole manuscript, figures, tables, and Supporting Information. The remaining “cytological endometritis” (S3, S4, S5, and S6 table captions) were replaced by “endometrial inflammation”. Figures 2, 4, and 5 were also modified to be consistent with the current nomenclature.

Additionally, it was observed that the S1 and S2 table captions in the manuscript did not fully match S1 and S2 table captions in the Supporting Information document. This issue was amended. In Tables S4 and S6, one header was modified to follow the same nomenclature (species detected) as in the main text.

Check that table and citation numbering are still correct after moving the former Table 3 to Supporting Information.

Following the editor’s comment, table numbering and citation in the text were carefully revised. There are 3 tables in the main manuscript, in the correct order and all cited in the text. Additionally, there are 6 tables in the Supporting Information document (S1-S6), all cited in the text in the correct order.

Reviewers’ comments

Reviewer #1: Thank you for taking the time to address my previous concerns.

The authors would like to thank the reviewer for the constructive feedback given in this review round. The manuscript has been modified according to the suggestions and a detailed response to each of the comments is provided below.

Minor comments

Adding Fig 2/3/4 into the main manuscript may help clarify the descriptive results for readers.

According to the journal’s submission guidelines, figures were uploaded as separate files. The figure captions were included in the main text following the citation (Fig 1, Fig 2…). The figures will be added to the main text during layout if the manuscript is accepted.

I can’t find anywhere a mention that in table 1 more mares with negative EI on UL had positive bacterial growth (30.5%) compared to those with no bacteria (23.8%). If you are including this table you should clarify this point. Line 313/314 seems to contradict this.

The authors would like to thank the reviewer for this observation. We agree that the descriptive frequencies presented in Table 1 may appear contrary to the main reported results when considered in isolation. However, Table 1 is intended only to summarize the raw distribution of cytology and culture results, and does not account for the effects of the number of bacterial isolates or the random-effects structure included in the generalized mixed-effects models. The statement in the discussion (L313-314) refers to the adjusted probabilities estimated by the GLMMs rather than to the descriptive percentages shown in Table 1. To avoid this potential misunderstanding, we have clarified this issue in the discussion (L327-330) to make it clear that conclusions are based on the model outputs and should not be drawn from the descriptive data in Table 1.

Table 2 is still confusing and I feel it could be represented in a clearer way.

The authors thank the reviewer for this comment, as the interpretation of the tables is ultimately important. We agree that the amount of information presented in this table, as well as is Table 3, requires careful interpretation. Both tables summarise the predicted probabilities obtained from the GLMMs for each bacterial species according to the number of isolates, together with the corresponding sample size and statistical significance. We considered alternative layouts, but none would improve the presentation of the results. Therefore, we retained the current format to ensure consistency between US and UL analyses. However, we modified the table caption (L259-261) to add the following clarification: “For each bacterial species, the table shows the predicted probability (± SE) of EI according to the number of bacterial isolates and the presence (Yes) or absence (No) of the species within each isolate category”. This change was also applied to Table 3 caption (L306-308). Additionally, the header “Bacteria isolated” was changed to “Species detected”.

Line 49: Consequently BACTERIAL/INFECTIONS endometritis diagnostic accuracy…

The manuscript was modified according to the reviewer’s suggestions (L49).

Line 55: Add reference for double guarded being less prone to contamination

Following the reviewer’s suggestion, the following reference was added (L57)

Line 105: Study could have been conducted at the farm level so worth stating this in limitations of the study

The authors would like to thank the reviewer for this observation. Although this is an interesting point, the objective of this study was to evaluate associations between bacteriological and cytological findings at the level of the individual uterine sample. Consequently, the sample was the appropriate experimental unit. Farm was included in the statistical models as a random effect to account for clustering of observations within farms. We believe that aggregating data at the farm level would have addressed a different biological question and would not have been appropriate for the purpose of the present study.

Line 342: Proportion of PMNs to epithelial cells may be more sensitive/specific

The manuscript was modified according to the reviewer’s suggestions (L358).

Reviewer #2: The manuscript has been throughly reviewed by 3 independent reviewers who all raised very serious concerns regarding the experimental protocol and the data analysis. I cannot say that I am impressed how the authors handled this revision. The reviewers' concerns have mainly been rebutted but there are no considerable changes in the manuscript. This reviewer is also still not convinced that the manuscript really fits into the scope of this journal.

The authors would like to thank the reviewer for their careful assessment of the revised manuscript and the constructive feedback.

Following the comments received during the previous review round, we carefully revised the manuscript to improve its clarity, transparency, and interpretation. One of the most important modifications was the replacement of the term cytological endometritis with endometrial inflammation, as appropriately recommended. This change reflects more accurately the nature of the data available and refines the interpretation of the study findings throughout the manuscript.

Several additional comments concerned limitations inherent to the retrospective nature of the study, including the absence of individual mare-level information and aspects of the laboratory sampling and processing protocols. We acknowledge these limitations and have expanded the Discussion to address their potential impact on the interpretation of the results. However, these aspects are intrinsic to the laboratory database o which the study is based and cannot be modified retrospectively.

Despite these limitations, we believe that the study provides valuable evidence derived from routine clinical practice. The data originate from samples collected by experienced equine practitioners under field conditions and processed by a reference diagnostic laboratory as part of its clinical service. Consequently, the findings reflect real-world veterinary practice and provide evidence that can directly support the interpretation of bacteriological and cytological results, contributing to clinical decision-making and proper antimicrobial use.

Regarding the suitability of the manuscript for PLOS ONE, we respectfully believe that the study falls within the journal’s stated scope. PLOS ONE considers original research across more than 200 subject areas, including the natural sciences and medicine, with primary research that contributes to the base of scientific knowledge. Our study presents original observational research based on a large retrospective dataset, applies appropriate statistical methodology, and provides evidence to improve the interpretation of bacteriological and cytological findings in equine reproductive medicine. We therefore believe that the manuscript meets the journal’s publication criteria and contributes to the body of scientific knowledge in this field. Additionally, we value the journal’s commitment to making research openly accessible, ensuring that findings are available to everyone interested regardless of institutional access.

Attachment Submitted filename: Response to Reviewers_R1.odt

# Editor report: Decision Letter 2

**DOI:** 10.1371/journal.pone.0356822.r005

**Authors:** Dinesh Dadarwal

10 Aug 2026

Evidence-based interpretation of uterine cultures in mares: linking bacterial growth patterns to endometrial inflammation

PONE-D-26-08838R2

Dear Dr. Yanez,

We’re pleased to inform you that your manuscript has been judged scientifically suitable for publication and will be formally accepted for publication once it meets all outstanding technical requirements.

Within one week, you’ll receive an e-mail detailing the required amendments. When these have been addressed, you’ll receive a formal acceptance letter and your manuscript will be scheduled for publication.

An invoice will be generated when your article is formally accepted. Please note, if your institution has a publishing partnership with PLOS and your article meets the relevant criteria, all or part of your publication costs will be covered. Please make sure your user information is up-to-date by logging into Editorial Manager at Editorial Manager® and clicking the ‘Update My Information' link at the top of the page. For questions related to billing, please contact billing support.

If your institution or institutions have a press office, please notify them about your upcoming paper to help maximize its impact. If they’ll be preparing press materials, please inform our press team as soon as possible -- no later than 48 hours after receiving the formal acceptance. Your manuscript will remain under strict press embargo until 2 pm Eastern Time on the date of publication. For more information, please contact onepress@plos.org.

Kind regards,

Dinesh Dadarwal

Academic Editor

PLOS One

Additional Editor Comments (optional):

Thank you for submitting the revised version of your manuscript that addresses comments from all reviewers.

Reviewers' comments:

# Editor report: Acceptance letter

**DOI:** 10.1371/journal.pone.0356822.r006

**Authors:** Dinesh Dadarwal

PONE-D-26-08838R2

PLOS One

Dear Dr. Yanez,

I'm pleased to inform you that your manuscript has been deemed suitable for publication in PLOS One. Congratulations! Your manuscript is now being handed over to our production team.

At this stage, our production department will prepare your paper for publication. This includes ensuring the following:

* All references, tables, and figures are properly cited

* All relevant supporting information is included in the manuscript submission,

* There are no issues that prevent the paper from being properly typeset

You will receive further instructions from the production team, including instructions on how to review your proof when it is ready. Please keep in mind that we are working through a large volume of accepted articles, so please give us a few days to review your paper and let you know the next and final steps.

Lastly, if your institution or institutions have a press office, please let them know about your upcoming paper now to help maximize its impact. If they'll be preparing press materials, please inform our press team within the next 48 hours. Your manuscript will remain under strict press embargo until 2 pm Eastern Time on the date of publication. For more information, please contact onepress@plos.org.

You will receive an invoice from PLOS for your publication fee after your manuscript has reached the completed accept phase. If you receive an email requesting payment before acceptance or for any other service, this may be a phishing scheme. Learn how to identify phishing emails and protect your accounts at https://explore.plos.org/phishing.

If we can help with anything else, please email us at customercare@plos.org.

Thank you for submitting your work to PLOS One and supporting open access.

Kind regards,

PLOS ONE Editorial Office Staff

on behalf of

Dr. Dinesh Dadarwal

Academic Editor

PLOS One
